# Do the Drivers of Food Choice Differ Between Healthier and Less Healthy Foods? A Systematic Review of Reviews

**DOI:** 10.1093/nutrit/nuaf202

**Published:** 2025-11-10

**Authors:** Bethany Parkes, Nia Morrish, Emma Frew, Laura Cornelsen, Richard Smith, Antonieta Medina-Lara

**Affiliations:** Department of Public Health and Sports Science, University of Exeter, Exeter EX2 4TH, United Kingdom; Department of Public Health and Sports Science, University of Exeter, Exeter EX2 4TH, United Kingdom; Centre for Economics of Obesity, Institute of Applied Health Research, University of Birmingham, Birmingham B15 2TT, United Kingdom; Population Health Innovation Lab, Department of Public Health, Environments and Society, London School of Hygiene & Tropical Medicine, London WC1H 9SH, United Kingdom; Department of Public Health and Sports Science, University of Exeter, Exeter EX2 4TH, United Kingdom; Department of Public Health and Sports Science, University of Exeter, Exeter EX2 4TH, United Kingdom

**Keywords:** review, drivers, food choice

## Abstract

**Context:**

This review is intended to fill gaps in reported literature reviews on food choices by performing a comprehensive overview of the similarities and differences in the drivers of more and less healthy food choices reported in published reviews.

**Objective:**

We sought to examine similarities and differences in drivers of healthier vs less healthy food choices in the reported literature on food choices.

**Data Sources:**

Searches were conducted in the Ovid (APA PsycINFO, Embase, MEDLINE), Econlit, and Scopus databases. A systematic review of reviews was undertaken according to the Preferred Reporting Items for Systematic reviews and Meta-Analyses (PRISMA) guidelines.

**Data Extraction:**

Reviews with samples of adults from high-income countries were eligible. Included reviews were appraised using the Critical Appraisal Skills Programme (CASP) checklist for systematic reviews, and the results were synthesized narratively. Original searches yielded 4887 results from which 102 full-text articles were screened. Updated searches in March 2024 led to a total of 59 included reviews.

**Data Analysis:**

Synthesized findings from included reviews were grouped into overarching categories of drivers and described narratively. Drivers of both less healthy and healthier food choices were income, availability, accessibility, social norms, health status, and the food environment. In contrast, some factors were reported primarily in relation to healthier or less healthy food. Being health conscious and motivated, having nutrition knowledge, and female gender were reported in relation to choices of healthier foods whereas price and/or affordability, stress, lack of time, and need for convenience were reported as drivers of choices of less healthy foods. The most common concern identified from the CASP checklist was a lack of quality appraisal among reviews.

**Conclusions:**

In this review we identified differences between drivers of food choice for healthier and less healthy food, indicating a possibility for policy interventions to target inappropriate drivers if this difference is not recognized. Rather, interventions targeting increasing healthier (or decreasing less healthy) food consumption may be more effective when targeting those relevant drivers.

**Systematic Review Registration:**

*PROSPERO registration No.* CRD42022363956.

## INTRODUCTION

Diet is an important modifiable lifestyle factor linked to the development of noncommunicable diseases, including type 2 diabetes and obesity as well as mental health disorders.^1–3^ Global diets are characterized by suboptimal intake of healthy foods (eg, whole grains) and overconsumption of unhealthy foods (eg, processed and red meat). Poor diet contributed to 11 million deaths globally in 2017 and more significantly affected areas with low incomes.^4^ Among European countries, there is a rising trend of obesity, with an expected obesity prevalence by 2025 of 20% or above for 33 of 53 European region countries, and even higher prevalences for some countries (eg, 34% for England).^5^ This trend is also predicted to occur in other high-income countries such as Australia^6^ and the United States.^7^

“Healthier” and “less healthy” foods and diets can be defined in different ways. In this study, the definition used for a healthier diet is a diet that mostly comprises “consuming core food groups recommended by population guidelines.”^8^ In this case, the guidelines used were those reported in the United Kingdom publication *The Eatwell Guide*.^9^ Meanwhile, the adopted definition of a less healthy diet was a diet that included high levels of consumption of “foods and drinks that are energy dense, nutrient poor and/or ultra-processed, comprising those with high levels of added sugar, saturated fats, and/or salt.”^10^ These definitions were chosen due to their alignment with national guidelines and were similar to those of other high-income countries, including the United States,^11^ Australia,^12^ and New Zealand.^13^ Overall diets, rather than individual food choices, were a focus of consideration due to their association with health outcomes and evidence suggesting that current dietary patterns do not adhere to national recommendations.^14–16^ Nevertheless, in this review we followed a broad approach inclusive of research on dietary patterns and food choices, recognizing that individual choices inform the overall diet.

Developing effective public health and policy interventions that can promote healthier eating and reduce less healthy eating requires understanding of what drives food choice. Price is one driver deemed to be important, with healthier foods judged as more expensive than less healthy foods,^17^ making price a key focus of policy.^18^ Policy interventions encouraging a shift toward healthier food consumption have sought to impact drivers, including marketing restrictions on less healthy food, labeling to provide nutrition information on foods, and restriction of availability. While fiscal and multicomponent interventions show the most promise, these have yet to yield major impacts in reducing population levels of overweight and obesity.^18–20^ With a need to improve efforts to support population dietary changes, a more granular understanding of food choice drivers may be useful.

A large number of drivers of food choice have been proposed in the literature. These include food context, resources, sensory properties, demographic factors, and physical and social environments.^21–23^ However, much of the current evidence often fails to consider the types of food associated with particular drivers and instead is based on the assumption that drivers are the same for healthier and less healthy food choices. Indeed, evidence suggests that there may be different motivations for eating different types of food, eg, Bell et al.^24^ found that nutrition and food safety were drivers of food consumption for fruits and vegetables, while taste was a driver for snack food consumption. Additionally, there is some evidence that drivers such as nutrition knowledge are associated with decreases in less-healthy eating, but not with increases in healthier eating.^25^ This finding suggests that drivers of healthier vs less healthy food choices may not be symmetrical in effect. While a number of reviews have explored drivers influencing healthier^8^^,^^26^ and less healthy eating,^27^ there is a need to compare and contrast this literature to understand where there may be differences between drivers of healthier and less healthy food choices. There has been 1 recent study in older adults (aged 65+ years) that reviewed drivers of healthy and unhealthy food choices,^28^ but a major gap remains in exploring food drivers for the wider adult population.

To address this gap, the primary research focus of this review of reviews was to examine similarities and differences in drivers for healthier versus less healthy choices. This focus provides a more nuanced understanding not only of why people eat what they eat but why people eat what they eat for different types of food (in this case, healthier and less healthy foods). Secondary research questions were (1) what food types have, or have not, been examined, to understand whether these food types show sub-differences in drivers, and (2) to what extent price and/or cost does or does not feature as a factor influencing food choice in comparison to other factors; and does this differ between healthier and less healthy food choices?

## METHODS

### Search Strategy

This review was performed according to the Preferred Reporting Items for Systematic reviews and Meta-Analyses (PRISMA) guidelines,^29^ and the protocol was registered in PROSPERO (CRD42022363956) (see Supplementary Materials 1d and 1e for checklists). A review of reviews was deemed to be the most appropriate method as the aim was to understand overarching drivers of food choice and dietary patterns across a wide body of literature, and due to the substantial number of existing reviews.

Key word searches were conducted in APA PsycINFO (Ovid), Embase (Ovid), MEDLINE (Ovid), Econlit (Ebsco), and Scopus in October 2022, with updated searches carried out in March 2024 to capture up-to-date evidence. Search terms related to drivers were combined with terms for “healthy” and “unhealthy” food to identify studies on food drivers (see Supplementary Materials 1a and 1b). These terms were then combined with search terms for different types of reviews. The strategy was developed with the support of an information specialist (S.R.) and was intended to take a broad perspective, with no specific types of healthier or less healthy foods set out. After initial searches, backward and forward citation chasing of included reviews was undertaken to identify any further relevant articles.

### Inclusion and Exclusion Criteria

Included reviews were required to have a focus on adults (minimum 50% of primary article samples), as they have agency over their food choices, and on individuals with no specific dietary requirements. Included articles were to also report studies on any drivers of healthier or less healthy food choices or diets, including food purchasing or consumption (referred to throughout as food choice). Review articles were included regardless of whether they defined the food or dietary patterns they examined, and for articles in which specific foods or food groups were stated, these were assessed against the reported definitions set out for “healthier” and “less healthy” food (see Introduction). For articles in which inclusion was unclear, the review team discussed these on a case-by-case basis.

Included populations were from high-income countries^30^ to ensure similar macro-economic factors for cross-study comparison. Only reviews from 2012 onward were included to reflect up-to-date research from the last 10 years (from initial searches). Reviews centered on interventions (eg, fiscal or weight loss programs), or conducted in restricted food environments (eg, cafeteria) were excluded as these were deemed to interfere with usual drivers of food choice. Descriptive accounts of dietary intake were also excluded. All included reviews were required to identify a clear research question and search strategy, and were restricted to English language (**Table 1**).

**Table 1.** Inclusion and Exclusion Criteria with PICOS

Type	Inclusion criteria	Exclusion criteria
PICOS		
Population	Adults from high-income countries with agency over their food choices who do not have any specific dietary requirements.	Populations from low- and middle-income countries. Populations with specific dietary needs, eg, athletes or those who do not have agency over their choices, eg, children. Reviews of settings in which food choices may be limited, eg, food choices in a cafeteria.
Intervention/ Exposure	A range of variables influencing food choice.	NA
Comparator	NA	NA
Outcome (driver types)	Drivers of any food purchasing/consumption.	Descriptive accounts of dietary intake.
Study design	Reviews with a clear research question and search strategy with named databases and inclusion/exclusion criteria.	Reviews of interventions to modify food choice.
Other		
Date	Last 10 years.	Articles published before 2012.
Language	English language.	Non-English articles.
Type of food	Reviews of healthier, or less healthy food choices.	Studies of drivers of food choice that do not distinguish between healthier and less healthy foods.

Abbreviation: NA, not applicable.

### Data Screening and Extraction

Screening was carried out in 2 stages, title and abstract screening followed by full-text screening, by 2 independent reviewers (B.P. and N.M.) using the criteria set out above. Duplicates were identified and removed. An updated search was carried out by B.P. and articles identified for possible inclusion were checked by N.M. At all stages of the searches, any disagreements were resolved by discussion with a third reviewer (A.M.L.). Endnote 20 and Microsoft Excel were used to store citations and record decisions.

Data extraction was carried out by B.P. using Microsoft Excel. Extracted data included main study characteristics, year of publication, quality assessment, details of primary articles (number of primary articles, sample size, country, participants, study design), and synthesized key findings of the reviews. Findings were extracted as drivers or barriers to healthier and less healthy food choices. Here, “drivers of food choice” included any factors encouraging increased or decreased consumption, and “barriers” indicated obstacles to consumption.

Study characteristic data were reviewed by N.M. for all included reviews after the initial screening, and for 10% of reviews included after the updated search. Main findings were reviewed by A.M.L. Any disagreements were resolved through discussion with all three reviewers. Where reviews lacked information about countries and/or age of participants,^31–38^ primary articles included in these reviews were checked to ensure a minimum of 50% were based on high-income countries and adult samples.

### Quality Assessment

Included reviews were assessed for risk of bias using the Critical Appraisal Skills Programme (CASP) checklist for Systematic Reviews.^39^ All reviews were assessed by B.P., with a randomly selected subset (10%, *n* = 6 reviews) assessed by N.M.. Results were presented using traffic light plots.

### Data Synthesis

Synthesized key findings of drivers and barriers influencing healthier and less healthy food choice were extracted from reviews. Drivers of healthier food choices were grouped and categorized into new overarching themes to elucidate common drivers across reviews. This process was carried out in Microsoft Excel by B.P. in the first instance, which was first reviewed by A.M.L., followed by all authors. A similar process was carried out for drivers of less healthy food choices and barriers to healthier food choices. This method was chosen due to its suitability given the mixed designs of included reviews. For the secondary research questions (1), reviews considering particular food types were compared to understand which drivers and barriers were reported in these reviews. Similarly, the secondary research question (2) led to a particular focus on how financial drivers were reported among reviews.

## RESULTS

**Figure 1.**
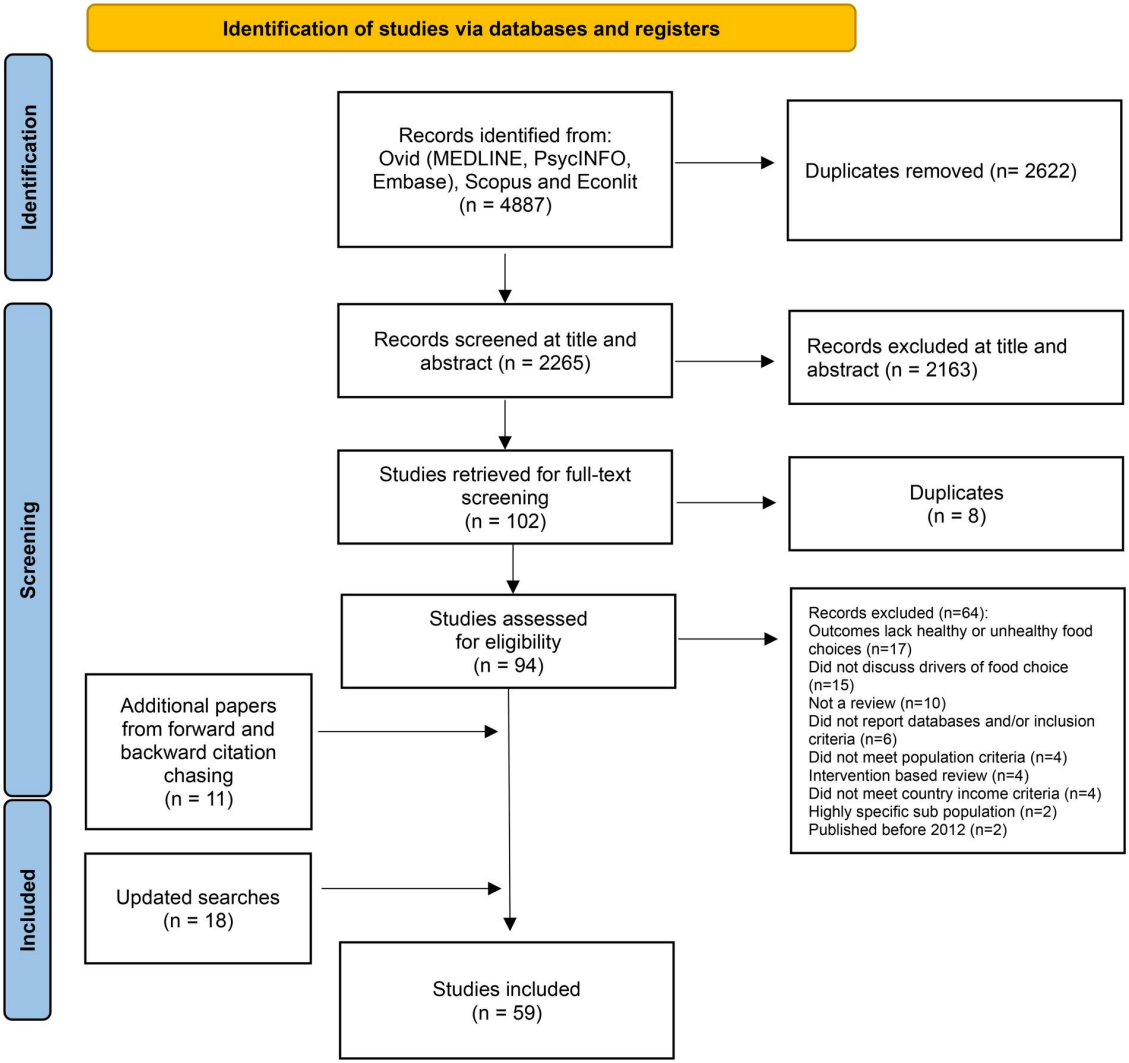
PRISMA (Preferred Reporting Items for Systematic Reviews and Meta-Analyses) Flow Chart of Screening Process

The searches yielded 4887 hits, of which 2265 remained once duplicates were removed. Overall, 102 studies were included following title and abstract screening, and were 30 included at full-text review. Forward and backward citation chasing yielded a further 11 studies for inclusion, bringing the total to 41 included reviews. Updated searches yielded a further 2448 hits, which together with forward and backward citating chasing led to a further 18 reviews being included (total = 59) (Figure 1).^29^

### Study Characteristics

Study characteristics are presented in **Table 2**.^8^^,^^26^^,^^28^^,^^31–38^^,^^40–87^ The included reviews were published between 2012 and 2023. The 59 reviews contained between 4 and 121 primary articles, of which the earliest was published in 1972 and the latest in 2023. Most reviews (78%, 46/59) spanned multiple continents, while 5 (8%) focused on a single country, and 8 (14%) failed to report details on geography for all included studies.

**Table 2.** Study Characteristics

**Author and** **year of publication**	Number and years of primary articles	Quality assessment	Countries of primary articles	Study design of primary articles	Participants of Primary Articles
Alsubhi et al. (2023)^40^	N = 15 articles. 2007 to 2020.	ISPOR Good Research Practices for Conjoint Analysis checklist.	Europe, North America, South America, Oceana, Asia.	Quantitative (stated preference) = 14. Quantitative (revealed preference) = 1.	Age: 10 adult samples, 3 unclear, 1 under 18 sample, 1 study did not use participants. Gender: not reported. Sample size: range 100 to 1211 participants.
Baker et al. (2022)^41^	N = 75 articles. 2000 to 2020.	NR	Europe, Asia, North America, South America, Australia and Oceania.	Survey = 52. Choice experiment = 5. Conjoint study = 4. Contingent valuation = 1. Experimental auction = 5. Experimental study = 5. Mixed methods = 2. Reanalysed existing data = 1.	Age: 18+ y. Gender: NR. Sample size: range 50–2392.
Barlow et al. (2016)^31^	N = 41 articles. 2000 to 2015.	Risk of bias evaluated.	Not reported	Cross sectional = 25. Experimental = 13. Longitudinal = 3.	Age: 4 child and adolescent samples, 35 adult samples (age not reported), 2 unreported. Gender: 14 female only samples, others NR. Sample size: Range 14 to 63 950 participants.
Bennett et al. (2022)^42^	N = 49 articles. 1979 to 2021.	Quality assessment.	Asia, Europe, North America, Africa, Oceana.	Quantitative (cross-sectional) = 44. Quantitative (longitudinal) = 2. Qualitative = 3	Age: 18+ years (1 sample is 16+, 4 samples are unknown). Gender: 31 mixed samples, 12 unknown, 5 all female samples, 1 all male sample. Sample size: range 25 to 186 916 participants.
Bernardo et al. (2017)^43^	N = 37 articles. 1994 to 2016.	NR	North America, Europe, Africa, South America, Asia.	Cross sectional = 33. Pre-test/ post-test = 1. Cross section of a longitudinal study = 1. Longitudinal = 1. Time series analysis = 1.	Age: University students. Gender: 31 mixed samples, 5 all female samples, 1 unreported. Sample size: Range 36–8516 participants.
Bimbo et al. (2017)^35^	N = 42 articles. 1999 to 2013.	Quality assessment.	Northern Europe, North America and Uruguay account for largest proportion of articles.	Single cross sectional = 26. Multiple cross-sectional = 8. Cohort = 1. Longitudinal = 2. Exploratory design (focus group) = 5.	Age: NR. Gender: NR. Sample size: Range 21 to 7947 participants.
Bivoltsis et al. (2023)^44^	N = 36 articles. 2005 to 2021.	Adapted quality assessment.	Oceana	Quantitative (longitudinal) = 9. Quantitative (cross-sectional) = 27.	Age: 30 adult samples, 5 samples under 18, 1 mixed age sample. Gender: 8 female samples, 28 not reported. Sample size: range 10 to 15 229 participants.
Caruso et al. (2023)^45^	N = 35 articles. 2005 to 2021.	JBI critical appraisal.	North America, Oceana, Europe, South America, Asia	Quantitative (cross-sectional) = 22. Quantitative (quasi-experimental) = 2. Quantitative (longitudinal) = 1. Qualitative (focus groups) = 5. Qualitative (interviews) = 3. Qualitative (focus groups and interviews) = 2.	Age: Mean age 18.1–27.5 years. Gender: 34 mixed samples, 1 not reported. Sample size: range 8 to 2665 participants.
Caso & Vecchio, (2022)^28^	N = 37 articles. 2010 to 2021.	NR	North America, Europe, Asia, South America, Oceania.	Quantitative (questionnaire) = 16. Qualitative (interviews) = 9. Mixed = 4. Qualitative (focus groups) = 3. Qualitative (mixed) = 3. Quantitative (mixed) = 1. RCT = 1.	Age: Independent older adults 65+. Gender: 33 mixed, one all female sample and one all male sample, 2 NR. Sample size: range12 to 83 364 participants.
Caspi et al. (2012)^46^	N = 38 articles. 1991 to 2011.	NR	North America, Europe, Oceania, Asia.	Cross-sectional = 35. Pre-post or natural design = 3.	Age: 31 adult samples, 5 children, 1 youth and adults, 1 unclear. Gender: 7 female samples, 1 male, rest NR. Sample size: range 102 to 836 281 participants (not all reported).
Cheong et al. (2022)^47^	N = 12 articles. 2008 to 2020.	Quality assessment.	North America, Oceana, Europe, Africa	Qualitative (focus groups and/or interviews) = 10. Qualitative (survey) = 2.	Age: working nurses. Gender: not reported. Sample size: range 8 to 264 participants.
Christenson et al. (2017)^48^	N = 14 articles. 2005 to 2015.	Critical appraisal assessment.	Australia.	Cross-sectional = 9. Focus groups = 3. Mixed methods = 2.	Age: range 18–75+ years. Gender: Majority female (not reported for each primary articles). Sample size: 29 to 3629 participants.
De Cianni et al. (2023)^33^	N = 31 articles, 1 conference poster. 2005 to 2022.	NR	North America, South America, Asia, Europe, Africa.	Empirical studies = 31. Conference poster = 1.	Age: NR. Gender: NR. Sample size: NR.
De Steur et al. (2015)^34^	N = 19 articles. 2002 to 2014.	NR	North America, Asia, Europe, South America, Oceania.	Survey = 7. Experiment = 8. Interview = 3. (not all reported)	Age: unknown. Gender: unknown. Sample size: range 100 to 2937 participants.
Devonport et al. (2019)^49^	N = 29 articles. 2004 to 2015.	Quality assessment.	North America, Europe, Asia.	Experimental = 19. Cross-sectional = 7. Interview = 1. Pre-post test = 1. Mixed design = 1.	Age: 18+years. Gender: 10 mixed samples, 18 all female, 1 not specified. Sample size: Range 16 to 761 participants.
Enriquez & Archila-Godinez, (2022)^37^	N = 51 articles. 1986 to 2020. Unclear if this is a complete list.	Not reported.	Not reported.	Not reported.	Not reported.
Eskandari et al. (2022)^50^	N = 47 articles. 2002 to 2021.	Methodological validity and risk of bias assessed.	North America, Europe.	Cross-sectional = 36. Focus group = 5. Interviews = 3. Photovoice = 1. Participatory approach = 1. Ethnography = 1.	Age: 32 adult samples, 11 child or adolescent samples, 3 mixed child and adult samples. Gender: 8 articles were all female samples, rest were mixed or unreported. Sample size: 10 to 8333 participants.
Fuente Gonzalez et al. (2022)^51^	N = 45 articles. Unable to report date range of publications.	NR	North America, Europe, South America. (not all reported)	Cohort study = 4. Cross-sectional = 12. RCT = 6. Experimental = 5. Longitudinal =2. Observational =1. (not all reported)	Age: 26 adult samples, 4 child and adolescent samples, rest NR. Gender: 10 female samples, 4 mixed samples, 16 NR. Sample size: NR.
Govindaraju et al. (2022)^52^	N = 51 articles. 2001 to 2020.	NR	Europe, North America, Oceania, Asia.	Cross-sectional = 15. Cohort = 2. Observational = 2. Quantitative (unclear) = 6. Interview = 7. Qualitative (mixed) = 9. Focus groups = 6. Mixed methods = 4.	Age: 50+. Gender: 38 mixed samples, 4 men only samples, 4 women only samples. 5 NR. Sample size: 7 to 17 330 participants.
Govzman et al. (2021)^53^	N = 121 articles. 2008 to 2018.	NR	Europe, North America, Oceania.	Cross-sectional = 51. Prospective cohort = 31. (Not all reported)	Age: 106 adult samples, 4 child samples, 7 mixed adult and child, 4 unclear. Gender: 24 female samples, 4 male samples, 79 mixed male/female samples,14 unclear. Sample size: 15 to 476 108 participants.
Gupta et al. (2018)^54^	N = 22 articles. 1985 to 2016.	Quality assessment.	North America, Europe, Africa, Asia.	Cross-sectional = 13. Experimental = 7. Quasi-experimental =1. Case study =1.	Age: 18 years+. Gender: 2 female samples, 20 mixed male and female samples. Sample size: range 1 to 3929 participants.
Gupta et al. (2019)^55^	N = 62 articles. 1972 to 2018.	NR	South America, Asia, North America, Europe, Africa, Oceania.	Quantitative cross-sectional = 30. Quantitative longitudinal = 10. Qualitative cross-sectional = 14. Qualitative longitudinal = 3. Mixed methods cross-sectional = 4. Mixed methods longitudinal = 1.	Age: not reported, assumed to be mostly adults as population is shift workers. Gender: 37 samples had males and females, 13 male samples, 8 female samples and 7 samples not specified. Sample size: range 7 to 3500 participants (1 sample not reported). [65 articles reported across all tables]
Hanna & Collins, (2015)^56^	N = 41 articles. 1990 to 2014.	Quality assessment.	Europe, Oceana, North America, Asia.	Quantitative (cross-sectional) = 38. Quantitative (cohort) = 3.	Age: 1 sample 16+, 40 samples 18+. Gender: 34 mixed samples, 5 female samples, 2 not reported. Sample size: range 34 (participants) to 94 564 (households).
Hanna et al. (2023)^57^	N = 29 articles. 2005 to 2023.	Quality assessment.	Europe, Asia, North America, Oceana	Quantitative cross-sectional = 21. Quantitative longitudinal = 5. Qualitative focus groups and interviews = 3.	Age: 16 years+. Gender: 28 mixed samples, 1 female sample. Sample size: range 30 to 20 478 participants.
Hill et al. (2022)^32^	N = 54 articles. 1991 to 2019.	Quality assessment.	NR	Cross-sectional = 17. Experimental = 16. Observational = 4. Mixed designs = 6. Within-subject design = 4. Longitudinal = 2. Prospective = 2. Diary study = 2. Information not accessible = 1.	Age: range 18 to 80+. Gender: 23 articles were all female, 2 all male samples, 29 articles were mixed samples. Size: range 9 to 65 235 participants.
Host et al. (2016)^58^	*n* = 24 articles. 1996 to 2014.	Evidence hierarchy analysis.	Europe, North America, Oceania.	Cross-sectional = 6. Interviews = 7. Mixed designs = 6. Focus Groups = 3. Observational = 2.	Age: 50+ years. Gender: 16 mixed samples, 3 all male, 1 all female, 4 not specified. Sample size: Range 12 to 3200 participants.
Kazmierski et al. (2021)^59^	*n* = 30 articles. 2008 to 2019.	NR	US	Cross-sectional = 25. Longitudinal repeated-measures design = 2. Ecological momentary assessment = 1. Experimental manipulation = 2.	Age: 25 adult samples, 1 adolescent and adult sample, 3 child and adolescent samples. Gender: 6 female samples, 2 male samples, 12 mixed male and female samples. Size: range 9 to 5301 participants.
Khan & Pandey, (2023)^60^	*n* = 84 articles. 2009 to 2020.	Not reported.	US accounted for highest proportion (70%).	Quantitative (experimental) = 69. Quantitative (modelling) = 9. Quantitative (experimental and modelling) = 2. Mixed methods (experimental and qualitative) = 2. Mixed methods (modelling and qualitative) = 2.	Age: 67 student samples, 66 unknown (reported overall, not by study). Gender: not reported. Sample size: not reported.
Khoshghadam & Rajabi, (2024)^61^	*n* = 111 articles. 1993 to 2023.	Only journals with 30+ H-index included.	Majority North America.	Qualitative (interview) = 2. Qualitative (interview, focus group) = 1. Quantitative (experimental) = 80. Quantitative (survey) = 13. Quantitative (secondary data) = 2. Theoretical = 13.	Age: 136 (51%) were students (mean 25.2 years), 47 samples (mean 35.7 years), 3 samples with 50+ years, (266 total study samples). Gender: not reported. Sample size: not reported.
Kouritzin et al. (2023)^62^	*n* = 4 articles. 2004 to 2020.	Not done.	Europe, North America, Oceana, Africa	Quantitative cross-sectional = 2. Quantitative longitudinal = 2.	Age: 17+ years. Gender: 3 mixed samples, 1 female sample. Sample size: range 54 to 9417 participants.
Li et al. (2019)^63^	*n* = 77 articles. 1985 to 2016.	Risk of bias assessment.	Europe, North America, South America, Oceania, Asia, Africa.	Cross-sectional = 26. Prospective = 20. Experimental = 5. Longitudinal = 8. Mixed design = 6. Repeated cross-sectional = 5. RCT = 2. Duplicates = 5.	Age: adults (range not reported). Gender: 1 mixed male and female samples. Others NR. Size: range 59 to 2031 participants.
Li et al. (2022)^64^	*n* = 11 articles. 2008 to 2021.	Certainty of the evidence and quality assessment.	North America, South America, Europe, Oceania.	Cross sectional = 11.	Age: University students. Gender: not reported. Sample size: range 35–1954
Lyzwinski et al. (2018)^65^	*n* = 51 articles. 1981 to 2016.	NR	Europe, North America, Oceania, South America, Asia, Africa.	Cross-sectional = 34. Longitudinal = 13. Pre-post = 1. Prospective = 2. Repeated measures = 1.	Age: 17 to 65 years. Gender: 11 female samples., 39 mixed male and female samples, 1 male sample. Sample size: range 34 to 14 804 participants.
Madlala et al. (2023)^66^	*n* = 47 articles. 2006 to 2021.	Not done.	North America, Oceana, South America, Europe	Cross-sectional qualitative (focus groups and/or interviews) = 7. Qualitative (participatory) = 2. Cross-sectional (mixed methods) = 14. Cross-sectional = 21. Cohort = 2. Ecological study = 1.	Age: 18 years+. Gender: 10 female samples, 13 mixed samples, 19 not reported, 5 samples did not include people. Sample size: range 10 to 5611 participants (some recruited only stores).
Maillet & Grouzet, (2021)^67^	*n* = 51 articles. 2001 to 2018.	Quality assessment.	Asia, Europe, Oceania, South America, North America.	Longitudinal = 30. Cross-sectional = 9. Focus groups = 6. Interviews = 3. Qualitative (mixed) = 3.	Age: University students (mean 18–22 years). Gender: mostly mixed, 2 samples with <30% female and 18 samples with <30% male participants. Sample size: range 4 to 6638 participants.
Marko et al. (2023)^68^	*n* = 29 articles. 2015 to 2021.	Quality assessment.	North America, Oceana, Europe, Asia, South America	Quantitative (cross-sectional) = 15. Quantitative (descriptive multi-centre design) = 2. Quantitative (longitudinal) = 1. Qualitative (interviews) = 5. Qualitative (focus groups) = 1. Qualitative (survey) = 1. Qualitative (focus groups and interviews) = 1. Mixed methods = 3.	Age: working age nurses. Gender: 4 female samples, 25 majority female samples. Sample size: range 17 to 1080 participants.
Mazri et al. (2020)^69^	*n* = 36 articles. 1985 to 2019.	NR	Asia, North America, South America, Europe.	Cross-sectional = 29. Interventions = 2. Prospective studies = 2. Prospective randomised controlled studies = 1. Follow-up studies = 1. The 36 included articles represented 35 studies.	Age: 18+ years. Gender: 27 mixed male and female samples, 6 all female samples, 2 not stated. Sample size: Range 66 to 439 933 participants.
McDermott et al. (2015)^70^	*n* = 43 articles. 1991 to 2014.	NR	Asia, Africa, North America, Europe, Oceania, South America.	Cross-sectional = 24. Prospective = 9. Mixed design = 5. Longitudinal = 2. Experimental = 1. Mixed qualitative = 1. Repeated cross-sectional = 1.	Age: 16 child and adolescent samples., 25 adult samples, 2 samples unspecified. Gender: 37 mixed male and female samples, 2 male samples, 3 female samples, 1 not specified. Sample size: range 75 to 3859.
Munt et al. (2017)^26^	*n* = 34 articles. 2006 to 2015.	NR	North America, Oceania, Europe, Asia.	Focus groups = 10. Questionnaire = 7. Survey = 4. Interview = 2. Food diary and questionnaire = 2. Questionnaire and focus group = 2. Other mixed design = 7.	Age: 18–24 years. Gender: Majority participants female. Sample size: Range 25 to 2942 participants.
Needham et al. (2020)^38^	*n* = 60 articles. 2002 to 2018.	Not done.	Australia.	Observational (cross-sectional) = 41. Observational (longitudinal) = 5. Observational (ecological) = 2. Observational (case studies or census audits) = 9. Observational (mixed methods) = 3.	Age: not reported. Gender: not reported. Sample size: range 10 to 15 229 participants. Many studies did not have participants.
Nicholls et al. (2017)^71^	*n* = 26 articles. 2000 to 2015.	Quality assessment.	Europe, North America, Africa, Asia, South America.	Cross-sectional = 21. Interviews and focus groups = 5.	Age: not reported assumed to be adults, all nurses. Gender: 4 female only samples. 6 mixed samples, 16 unspecified samples. Sample size: range 7 to 8665 participants.
Ogundijo et al. (2022)^72^	*n* = 15 articles. 2002 to 2020.	Quality assessment.	UK.	Interviews = 3. Focus groups = 2. RCT intervention experiment = 3. Choice experiment = 2. Survey = 2. Mixed methods = 3.	Age: 18+ years. Gender: 1 female sample, 10 mixed samples, 4 unspecified samples. Sample size: range 8 to 2019- participants.
Ojo et al. (2023)^73^	*n* = 20 articles. 2004 to 2022.	Quality assessment.	Europe, North America, Oceana	Qualitative (focus groups) = 9. Qualitative (interviews) = 6. Qualitative (interviews and observations) = 5.	Age: 13 adult samples, 5 adolescent and adult samples, 2 not reported. Gender: 10 mixed samples, 5 female samples, 5 not reported. Sample size: range 12 to 83 participants.
Pinto et al. (2021)^74^	*n* = 60 articles. 2016 to 2020.	Quality assessment.	South America, North America, Asia, Africa, Oceania, Europe.	Focus Groups = 18. Interviews = 18. Focus Groups and other = 7. Other = 8. Word association = 4. Mixed = 3. Interview and other = 2.	Age: 4 child and adolescent samples, 3 mixed child and adult samples, 47 adult samples, 6 samples NR. Gender: 44 mixed male and female samples, 8 female samples and 8 unknown. Sample size: range 10–2381 participants.
Pitt et al. (2017)^75^	*n* = 30 articles. 2001 to 2015.	Quality assessment.	North America, Oceania, Europe, South America.	Focus Groups = 14. Interviews = 12. Mix of interviews and focus groups = 4.	Age: all adults, range NR. Gender: 18 mixed male and female samples, 9 female samples and 3 unknown. Sample size: range 14–186 participants.
Poggiogalle et al. (2021)^76^	*n* = 39 articles. 1982 to 2019.	Quality assessment.	Europe, North America, Oceania, Asia.	Cross-sectional = 37. Longitudinal = 2.	Age: 65+ years. Gender: 33 mixed, 4 female samples, 2 NR. Sample size: Range 29 to 28 566 participants.
Ravikumar et al. (2022)^77^	*n* = 14 articles. 2012 to 2019.	Quality assessment.	North America, Oceana, Europe	Qualitative (interviews) = 6. Qualitative (focus groups) = 3. Qualitative (photovoice) = 1. (not all reported)	Age: all parents, mean age 20–65 years (not all reported). Gender: 8 mixed samples, 5 female samples. Sample size: range 5 to 150 participants (not all reported).
Mello Rodrigues et al. (2019)^78^	*n* = 71 articles. 2009 to 2018.	NR	Europe, North America, South America, Asia, Africa, Oceania.	Cross sectional = 67. Mixed cross-sectional and longitudinal = 1. Micro longitudinal = 1. Time series analysis = 1. Retrospective survey = 1.	Age: mean 21.6 years. Gender: 61 samples mixed, 8 female samples, 1 male sample, 1 not stated. Sample size: Range 45 to 8516 participants.
Shahrin et al. (2019)^79^	*n* = 15 articles. 2007 to 2017.	NR	Africa, North America, Europe, Oceania, Asia.	Cross-sectional = 6. Observational = 1. Focus groups = 4. Qualitative (cross-sectional) = 1. Interview = 2. Qualitative (cohort) = 1.	Age: 50+ years, one study included participants under 50 years. Gender: NR. Sample size: range 12–98 733 participants.
Sobhani & Babashahi, (2020)^80^	*n* = 34 articles. 1998 to 2016.	Quality assessment.	Europe, North America, South America, Oceania, Asia.	Unclear.	Age: households. Gender: NR. Sample size: range 80–164 315 participants.
Teixeira et al. (2022)^81^	*n* = 43 articles. 2009 to 2021.	Quality assessment.	Europe, Asia, North America, South America.	Cross sectional = 36. Cohort = 5. Dual design (cross-sectional and interventional) = 1. Interventional = 1.	Age: 33 adult samples. 9 child and/or adolescent samples, 1 mixed child and adult sample. Gender: 6 female samples. 37 not stated. Sample size: range 72 and 5536 participants.
Tsofliou et al. (2022)^82^	*n* = 18 articles. 2012 to 2021.	Quality assessment.	Europe, North America, Oceana	Observational, cross-sectional = 12. Qualitative = 6.	Age: 17 adult samples, 1 unclear. Gender: 14 mixed samples, 3 female samples, 1 unclear. Sample size: range 11 to 36 032 participants
Turner et al. (2021)^83^	*n* = 36 articles. 2012 to 2020.	Quality assessment.	North America, South America, Asia, Europe, Oceania.	All cross-sectional.	Age: 18+years. Gender: 35 mixed male and female samples, 1 female samples. Sample size: range 77–102 869 participants.
van der Merwe et al. (2022)^84^	*n* = 24 articles. 2011 to 2020.	Quality assessment.	North America, South America, Asia, Europe.	Cross sectional = 20. Cohort study = 2. Randomised control trial = 1. Population-based study = 1.	Age: adults 18+ years. Gender: 21 studies mixed, 3 studies female. Sample size: range 44–3304 participants.
Vilar-Compte et al. (2021)^85^	*n* = 68 articles. 2000 to 2019.	Quality assessment.	Asia, Africa, North America, South America, Oceania, Europe. Not all reported	Cross-sectional = 44. Geospatial = 5. Longitudinal/panel = 3. Case-control = 1. Cohort = 2. Interviews = 5. Focus groups = 3. Ethnography = 2. Photovoice = 1. Qualitative (unclear) = 2.	Age: 13 child and/or adolescent samples, 25 adult samples, 7 mixed adult and child samples, 16 'household’ samples, 7 'other’. Gender: 9 female samples. 1 male, 58 unstated. Sample size: NR
Walker-Clarke et al. (2022)^86^	*n* = 53 articles. 2008 to 2018.	Quality assessment.	Europe, Oceania, Asia, North America, South America.	Cross-sectional = 26. Interviews = 12. Focus groups = 9. RCT = 5. Mixed methods = 1.	Age: 60+ years. Gender: NR. Sample size: range 10–82 364 participants.
Yamaguchi et al. (2022)^87^	*n* = 19 articles. 2010 to 2020.	Risk of bias assessed.	North America, Oceana, Asia, Europe	Observational cross-sectional = 18. Observational longitudinal = 1.	Age: 18+ years. Gender: 1 female sample, 18 not reported. Sample size: range 221 to 83 384 participants
Zanchini et al. (2022)^36^	*n* = 30 articles. 2002 to 2020.	NR	NR	Choice Experiment = 10. Cross-sectional = 9. Experimental = 9. Projective design = 1. Mixed design = 1.	Not reported
Zorbas et al. (2018)^8^	*n* = 39 articles. 2008 to 2017.	Quality assessment.	Oceania, North America, Europe.	Focus groups = 26. Interviews = 10. Photovoice = 2. Survey = 1	Age: 16+ years, not all reported. Gender: 26 mixed male and female samples, 6 female, 4 male samples, 3 unreported. Sample size: range 10–147 participants.

Abbreviations: NA, not applicable; NR, not reported.

While 15 reviews did not provide complete information, from the available data, over 60% (33/59) focused on adults only (age 18+ years), or included samples of individuals 16 to17 years old (6/59), while 13 reviews had participants of mixed age ranges; 1 review focused on households, and 6 reviews did not report on the age of study participants from primary articles. Forty reviews (68%) included primary articles which had both male and female samples, 15 reviews (25%) did not report on gender, four reviews (7%) reported only female participant samples. Ethnicity of study participants in primary articles were reported partially or fully in 14 reviews (24%).

Of the included reviews, 85% (50/59) had a mix of study designs among their primary articles. Five reviews synthesized qualitative research, 2 included primary articles with cross-sectional designs only, and 2 did not report the study designs of included articles. From the available information from reviews, the largest sample size covered by a primary article was 836 281 study participants^46^ and the smallest was just 1 study participant.^54^ Five reviews did not report any of the sample sizes of their included primary articles. Most reviews synthesized findings narratively (55/59), while a minority (4/59) also included meta-analysis.

The primary articles from included reviews were retrieved to assess for overlap. A primary article citation matrix was created and hand-searched for duplicates. A full list of primary article titles was not available or was unclear for 5 reviews.^34^^,^^37^^,^^51^^,^^53^^,^^61^ Among the included reviews, there were 1988 titles that were accessible, among which 242 were repeated at least once (12%).

#### Definitions of Healthier and Less Healthy Food

Overall, there was a lack of clarity regarding the parameters chosen by the review authors with respect to the classification of food into healthier and less healthy categories; only 37% (22/59) of reviews provided definitions relating to food. Seventeen of these reviews had clear definitions for healthier and/or less healthy food relevant to their review, and a further 5 of these reviews defined other food types, eg, functional food, seafood, etc. Among the 24 reviews which focused on particular food types, 16 provided definitions relating to that particular food type, or a definition for healthier or less healthy food or eating behavior.^8^^,^^31^^,^^34^^,^^36^^,^^40^^,^^41^^,^^47^^,^^51^^,^^53^^,^^61^^,^^63^^,^^68^^,^^71^^,^^72^^,^^82^^,^^85^ While providing food definitions within reviews was not an inclusion criterion, the lack of clarity in definitions across the literature was an unexpected but important finding of this review.

### Quality Appraisal

**Table 3** ^8^ ^,^ ^26^ ^,^ ^28^ ^,^ ^31–38^ ^,^ ^40–87^ shows the quality of the included reviews. The overall quality of the reviews was good, but at least 1 area of concern was identified in 26 (44%) reviews, with lack of quality appraisal being the most common criterion of concern (*n* = 25). Examining the precision of results was possible for 4 reviews in which meta-analysis was carried out.^32^^,^^50^^,^^63^^,^^70^ Most articles were deemed to have results that were generalizable to the population of interest of the review (*n* = 51), having clearly reported study participant ages and countries of primary articles. Five reviews lacked sufficient information about sources of included studies to enable an assessment regarding whether all important and relevant studies were included. This included a lack of detail about whether reference lists were followed up or whether gray literature was excluded, and 3 reviews for which literature was searched only from a single database.^37^^,^^46^^,^^60^^,^^61^^,^^79^

**Table 3.** CASP Quality Appraisal Checklist

Author and year	Did the review address a clearly focused question?	Did the authors look for the right type of articles?	Do you think all the important, relevant studies were included?	Did the review’s authors do enough to assess quality of the included studies?	If the results of the review have been combined, was it reasonable to do so?	How precise are the results?	Can the results be applied to the local population?	Were all important outcomes considered?	Are the benefits worth the harms and costs?
Alsubhi et al. (2023)^40^	Yes	Yes	Yes	Yes	Yes	NA	Yes	Yes	NA
Baker et al. (2022)^41^	Yes	Yes	Yes	No	Yes	NA	Yes	Yes	NA
Barlow et al. (2016)^31^	Yes	Yes	Yes	Yes	Yes	NA	Can’t Tell	Yes	NA
Bennett et al. (2022)^42^	Yes	Yes	Yes	Can’t Tell	Yes	NA	Yes	Yes	NA
Bernardo et al. (2017)^43^	Yes	Yes	Yes	No	Yes	NA	Yes	Yes	NA
Bimbo et al. (2017)^35^	Yes	Yes	Yes	Yes	Yes	NA	Can’t Tell	Yes	NA
Bivoltsis et al. (2023)^44^	Yes	Yes	Yes	Yes	Yes	NA	Yes	Yes	NA
Caruso et al. (2023)^45^	Yes	Yes	Yes	Yes	Yes	NA	Yes	Yes	NA
Caso & Vecchio, (2022)^28^	Yes	Yes	Yes	No	Yes	NA	Yes	Yes	NA
Caspi et al. (2012)^46^	Yes	Can’t Tell	Can’t Tell	No	Yes	NA	Yes	Yes	NA
Cheong et al. (2022)^47^	Yes	Yes	Yes	Yes	Yes	NA	Yes	Yes	NA
Christenson et al. (2017)^48^	Yes	Yes	Yes	Yes	Yes	NA	Yes	Yes	NA
De Cianni et al. (2023)^33^	Yes	Yes	Yes	No	Yes	NA	Can’t Tell	Yes	NA
De Steur et al. (2015)^34^	Yes	Yes	Yes	No	Yes	NA	Can’t Tell	Yes	NA
Devonport et al. (2019)^49^	Yes	Yes	Yes	Yes	Yes	NA	Yes	Yes	NA
Enriquez & Archila-Godinez, (2022)^37^	Yes	Can’t Tell	Yes	No	Yes	NA	Can’t Tell	Yes	NA
Eskandari et al. (2022)^50^	Yes	Yes	Yes	Yes	Yes	Yes^a^	Yes	Yes	NA
Fuente Gonzalez et al. (2022)^51^	Yes	Yes	Yes	No	Yes	NA	Yes	Yes	NA
Govindaraju et al. (2022)^52^	Yes	Yes	Yes	No	Yes	NA	Yes	Yes	NA
Govzman et al. (2021)^53^	Yes	Yes	Yes	No	Yes	NA	Yes	Yes	NA
Gupta et al. (2018)^54^	Yes	Yes	Yes	Yes	Yes	NA	Yes	Yes	NA
Gupta et al. (2019)^55^	Yes	Yes	Yes	No	Yes	NA	Yes	Yes	NA
Hanna & Collins, (2015)^56^	Yes	Yes	Yes	Yes	Yes	NA	Yes	Yes	NA
Hanna et al. (2023)^57^	Yes	Yes	Yes	Yes	Yes	NA	Yes	Yes	NA
Hill et al. (2022)^32^	Yes	Yes	Yes	Yes	Yes	Yes^b^	Can’t Tell	Yes	NA
Host et al. (2016)^58^	Yes	Yes	Yes	Yes	Yes	NA	Yes	Yes	NA
Kazmierski et al. (2021)^59^	Yes	Yes	Yes	No	Yes	NA	Yes	Yes	NA
Khan & Pandey, (2023)^60^	Yes	Can’t Tell	Can’t Tell	No	Yes	NA	Yes	Yes	NA
Khoshghadam & Rajabi, (2024)^61^	Yes	Yes	Can’t Tell	No	Yes	NA	Yes	Yes	NA
Kouritzin et al. (2023)^62^	Yes	Yes	Yes	No	Yes	NA	Yes	Yes	NA
Li et al. (2019)^63^	Yes	Yes	Yes	Yes	Yes	Yes^c^.	Yes	Yes	NA
Li et al. (2022)^64^	Yes	Yes	Yes	Yes	Yes	NA	Yes	Yes	NA
Lyzwinski et al. (2018)^65^	Yes	Yes	Yes	No	Yes	NA	Yes	Yes	NA
Madlala et al. (2023)^66^	Yes	Yes	Yes	No	Yes	NA	Yes	Yes	NA
Maillet & Grouzet, (2021)^67^	Yes	Yes	Yes	Yes	Yes	NA	Yes	Yes	NA
Marko et al. (2023)^68^	Yes	Yes	Yes	Yes	Yes	NA	Yes	Yes	NA
Mazri et al. (2020)^69^	Yes	Yes	Yes	No	Yes	NA	Yes	Yes	NA
McDermott et al. (2015)^70^	Yes	Yes	Yes	No	Yes	Yes^d^	Yes	Yes	NA
Munt et al. (2017)^26^	Yes	Yes	Yes	No	Yes	NA	Yes	Yes	NA
Needham et al. (2020)^38^	Yes	Yes	Yes	No	Yes	NA	Can’t Tell	Yes	NA
Nicholls et al. (2017)^71^	Yes	Yes	Yes	Yes	Yes	NA	Yes	Yes	NA
Ogundijo et al. (2022)^72^	Yes	Yes	Yes	Yes	Yes	NA	Yes	Yes	NA
Ojo et al. (2023)^73^	Yes	Yes	Yes	Yes	Yes	NA	Yes	Yes	NA
Pinto et al. (2021)^74^	Yes	Yes	Yes	Yes	Yes	NA	Yes	Yes	NA
Pitt et al. (2017)^75^	Yes	Yes	Yes	Yes	Yes	NA	Yes	Yes	NA
Poggiogalle et al. (2021)^76^	Yes	Yes	Yes	Yes	Yes	NA	Yes	Yes	NA
Ravikumar et al. (2022)^77^	Yes	Yes	Yes	Yes	Yes	NA	Yes	Yes	NA
Mello Rodrigues et al. (2019)^78^	Yes	Yes	Yes	No	Yes	NA	Yes	Yes	NA
Shahrin et al. (2019)^79^	Yes	Can’t Tell	Yes	No	Yes	NA	Yes	Yes	NA
Sobhani & Babashahi, (2020)^80^	Yes	Yes	Yes	Yes	Yes	NA	Yes	Yes	NA
Teixeira et al. (2022)^81^	Yes	Yes	Yes	Yes	Yes	NA	Yes	Yes	NA
Tsofliou et al. (2022)^82^	Yes	Yes	Yes	Yes	Yes	NA	Yes	Yes	NA
Turner et al. (2021)^83^	Yes	Yes	Yes	Yes	Yes	NA	Yes	Yes	NA
van der Merwe et al. (2022)^84^	Yes	Yes	Yes	Yes	Yes	NA	Yes	Yes	NA
Vilar-Compte et al. (2021)^85^	Yes	Yes	Yes	Yes	Yes	NA	Yes	Yes	NA
Walker-Clarke et al. (2022)^86^	Yes	Yes	Yes	Yes	Yes	NA	Yes	Yes	NA
Yamaguchi et al. (2022)^87^	Yes	Yes	Yes	Yes	Yes	NA	Yes	Yes	NA
Zanchini et al. (2022)^36^	Yes	Yes	Yes	No	Yes	NA	Can’t Tell	Yes	NA
Zorbas et al. (2018)^8^	Yes	Yes	Yes	Yes	Yes	NA	Yes	Yes	NA

Abbreviation: NA, not applicable.

a Main meta-analysis component showed a small but significant association between food insecurity and obesity; OR: 1.503, 95% CIs: 1.432–1.577, p-value = .000.

b Main meta-analysis component showed significant association between stress and food intake. Hedges’ g = 0.114, 95% CIs: 0.061–0.166, p<.001.

c Meta-analysis results reported in tables with confidence intervals and p values. Intention-choosing health promoting foods r_z_ = 0.46, 95% Cis: 0.39–0.53, *p* < 0.001.

d Variables in the theory of planned behavior had medium to large associations with intention and behavior. Attitude-intention r_+_ = 0.54, PBC-intention r_+_ = 0.42, subjective norm-intention r_+_ = 0.37, intention-behavior r_+_ = 0.45, PBC-behavior r_+_ = 0.27. Source: (CASP-UK.net).

### Drivers of Healthier and Less Healthy Food

The drivers were classified as drivers of healthier or less healthy food choice, or as barriers to healthier food. It is acknowledged that while the authors of this review recognized this distinction, included reviews may not have been written with this categorization in mind. Barriers to less healthy food choice were extracted but not synthesized due to the scarcity of results reported. Findings appearing across 2 or more reviews were categorized thematically, and overarching drivers are presented in **Table 4**  ^8^^,^^26^^,^^28^^,^^32–37^^,^^40–87^ (see Supplementary Materials 1c for the extracted detailed findings).

**Table 4.** Main Drivers of Healthier Food, Less Healthy Food, and Barriers to Food Choice

Category	Drivers of healthier food	Drivers of less healthy food	Barriers to heathier food
Demographic Drivers	Female gender^26^^,^^28^^,^^35^^,^^36^^,^^40^^,^^78^^,^^80^^,^^82^	Lower income^28^^,^^76^	Higher education^34^^,^^40^
	Higher education^28^^,^^36^^,^^37^^,^^42^^,^^53^^,^^76^^,^^80^^,^^82^		Lower income^50^^,^^82^
	Higher income^28^^,^^36^^,^^53^^,^^58^^,^^80^		Lower socioeconomic status^8^^,^^85^
	Higher occupational class^28^^,^^42^^,^^53^^,^^80^		Younger age^34^^,^^40^^,^^82^
	Higher socioeconomic status^53^^,^^80^		
	Older age^28^^,^^34–36^^,^^40^^,^^53^^,^^73^		
Environmental Drivers	Accessibility^66^^,^^83^	Accessibility^66^^,^^71^^,^^73^^,^^75^	Accessibility^26^^,^^50^^,^^58^^,^^62^^,^^66^^,^^68^^,^^82^
	Availability^46^^,^^53^^,^^66^^,^^71^^,^^83^	Availability^28^^,^^55^^,^^71^^,^^72^^,^^75^	Availability^8^^,^^26^^,^^45^^,^^48^^,^^50^^,^^53^^,^^64^^,^^66–68^^,^^73^^,^^77^^,^^82^
	Healthier food environment^44^^,^^87^	Food environment^28^^,^^62^^,^^64^^,^^77^	Food environment^66^^,^^67^
	Marketing^28^^,^^41^^,^^66^^,^^72^	Price/affordability^28^^,^^45^^,^^52^^,^^64^^,^^71^^,^^75^^,^^80^	Price / affordability^8^^,^^26^^,^^33^^,^^45^^,^^47^^,^^48^^,^^50^^,^^53^^,^^62^^,^^64^^,^^66–68^^,^^73^^,^^77^^,^^82^
			Lack of facilities ^26^^,^^47^^,^^67^
Household and Lifestyle	Habits^8^^,^^26^^,^^28^^,^^48^^,^^52^	Not living with parents^43^^,^^67^	Family preferences^48^^,^^50^^,^^53^
	Physical activity^34^^,^^36^^,^^40^^,^^53^^,^^58^	Time and convenience^45^^,^^77^	Time and convenience^8^^,^^26^^,^^62^^,^^67^^,^^73^^,^^77^^,^^82^
	Prior consumption^34^^,^^41^^,^^82^	Working hours^55^^,^^71^	Transportation^50^^,^^62^^,^^66^
	Seeking novelty^41^^,^^48^	Living alone and loneliness^56–58^	Working hours^47^^,^^55^^,^^68^^,^^71^
	Living with family or others^76^^,^^78^		Work break availability^47^^,^^55^
	Access to transport^58^^,^^66^		Reluctance to change^58^^,^^62^^,^^86^
	Marital status or cohabitation^76^^,^^82^		
Knowledge and Skills	Cooking skills^8^^,^^28^^,^^52^^,^^53^^,^^58^		Lack of cooking skills^26^^,^^53^^,^^73^^,^^82^
	Knowledge of functional food^36^^,^^41^		
	Nutrition knowledge^8^^,^^28^^,^^33^^,^^35^^,^^52^^,^^53^^,^^58^^,^^71^^,^^73^		
Physical Characteristics	Avoid ill-health^52^^,^^53^^,^^55^^,^^82^	Dental incapabilities^28^^,^^58^	Poor health or physical function^52^^,^^53^^,^^82^
	Benefits to self-image^26^^,^^73^^,^^82^	Evening chronotype^69^^,^^81^^,^^84^	
	Good Physical Health^28^^,^^58^	Low sensory perception^28^^,^^58^	
	Health problems^41^^,^^73^		
	Higher weight or BMI^36^^,^^40^^,^^41^		
	Morning chronotype^69^^,^^81^^,^^84^		
Product Characteristics	Food naturalness ^28^^,^^74^		Shelf-life^8^^,^^50^
	Health information^28^^,^^33^^,^^34^^,^^37^^,^^41^		Undesirable sensory attributes^8^^,^^26^^,^^45^^,^^53^^,^^74^^,^^82^
	Nutritional content^34^^,^^72^		
	Positive sensory attributes^28^^,^^33^^,^^41^^,^^48^^,^^82^		
	Traffic light labels^60^^,^^72^		
Psychological Drivers	Dietary control^63^^,^^70^^,^^86^	Consumption for alertness/ energy^55^^,^^71^	Competing priorities^47^^,^^62^
	Health and nutrition interest^28^^,^^58^	Emotional eating/ coping^49^^,^^51^^,^^61^^,^^71^	Lack of motivation^26^^,^^47^^,^^67^^,^^71^^,^^82^
	Health concerns^36^^,^^40^	Negative emotional states^37^^,^^60^^,^^86^	Stress^26^^,^^65^^,^^68^^,^^82^
	Health conscious and motivated^26^^,^^28^^,^^37^^,^^41^^,^^52^^,^^55^^,^^60^^,^^63^^,^^71^^,^^72^^,^^82^	Stress^32^^,^^49^^,^^51^^,^^55^^,^^59^^,^^65^	Unfamiliarity^33^^,^^41^
	Nutritional and health benefit beliefs^8^^,^^36^^,^^41^^,^^48^^,^^53^		Lack of self-control or regulation^26^^,^^47^
	Positive emotional states^28^^,^^53^^,^^78^^,^^82^^,^^86^		Lack of self-efficacy and confidence^47^^,^^48^^,^^71^
	Valuing health and healthy eating^26^^,^^58^^,^^78^^,^^79^		Negative perceptions and beliefs^41^^,^^50^^,^^53^^,^^82^
	Self-regulation^26^^,^^82^		
Social Drivers	Commensal eating^82^^,^^86^	Social norms^54^^,^^67^	Negative influence of others^26^^,^^47^^,^^55^^,^^67^^,^^82^
	Community, family and peer support^8^^,^^26^^,^^28^^,^^47^^,^^58^^,^^67^^,^^68^^,^^73^^,^^82^		Social norms of acceptability^8^^,^^26^^,^^82^
	Social norms^26^^,^^47^^,^^53^^,^^63^		

Frequently reported drivers of less healthy food choice are price and/or affordability (*n* = 7 reviews), experiencing stress (*n* = 6), and the availability of less healthy food alongside the lack of availability of healthier food (*n* = 5). Being health conscious and motivated (*n* = 11 reviews), nutrition knowledge (*n* = 9), community, family and peer support (*n* = 9), female gender (*n* = 8), higher education (*n* = 8) and older age (*n* = 7) are frequently reported drivers of healthier food choice. The most frequently reported barriers to healthier food choice are price and/or affordability (*n* = 16 reviews), availability, including both poor availability of healthy food and high availability of unhealthy food (*n* = 13), a lack of time and need for convenience (*n* = 8), difficulties accessing healthier food and high accessibility of less healthy food (*n* = 7), and undesirable sensory attributes of healthy food eg, taste, texture or smell (*n* = 6). Socioeconomic status is also identified as a barrier, in that lower socioeconomic groups are reported to experience greater barriers to healthier eating through poorer access to healthier foods, increased transportation challenges and higher prices of healthier foods in poorer neighbourhoods. Furthermore, the finding of higher education as a barrier may contradict expectation. This was reported to be associated with a lower willingness to pay for genetically modified foods with enhanced vitamin levels^34^ and healthier food products^40^ and may therefore not be applicable to healthier diets in general and this finding was noted to be contradictory to other research.^40^

Results should be interpreted carefully as some concepts may be overlapping. For example, the food environment appears separately as a driver (barrier) to availability and accessibility, due to differences in reporting of results among included reviews. Despite this, availability and accessibility have been described as elements of the food environment by some.^88^

#### Similarities and Differences in Drivers

**Figure 2.**
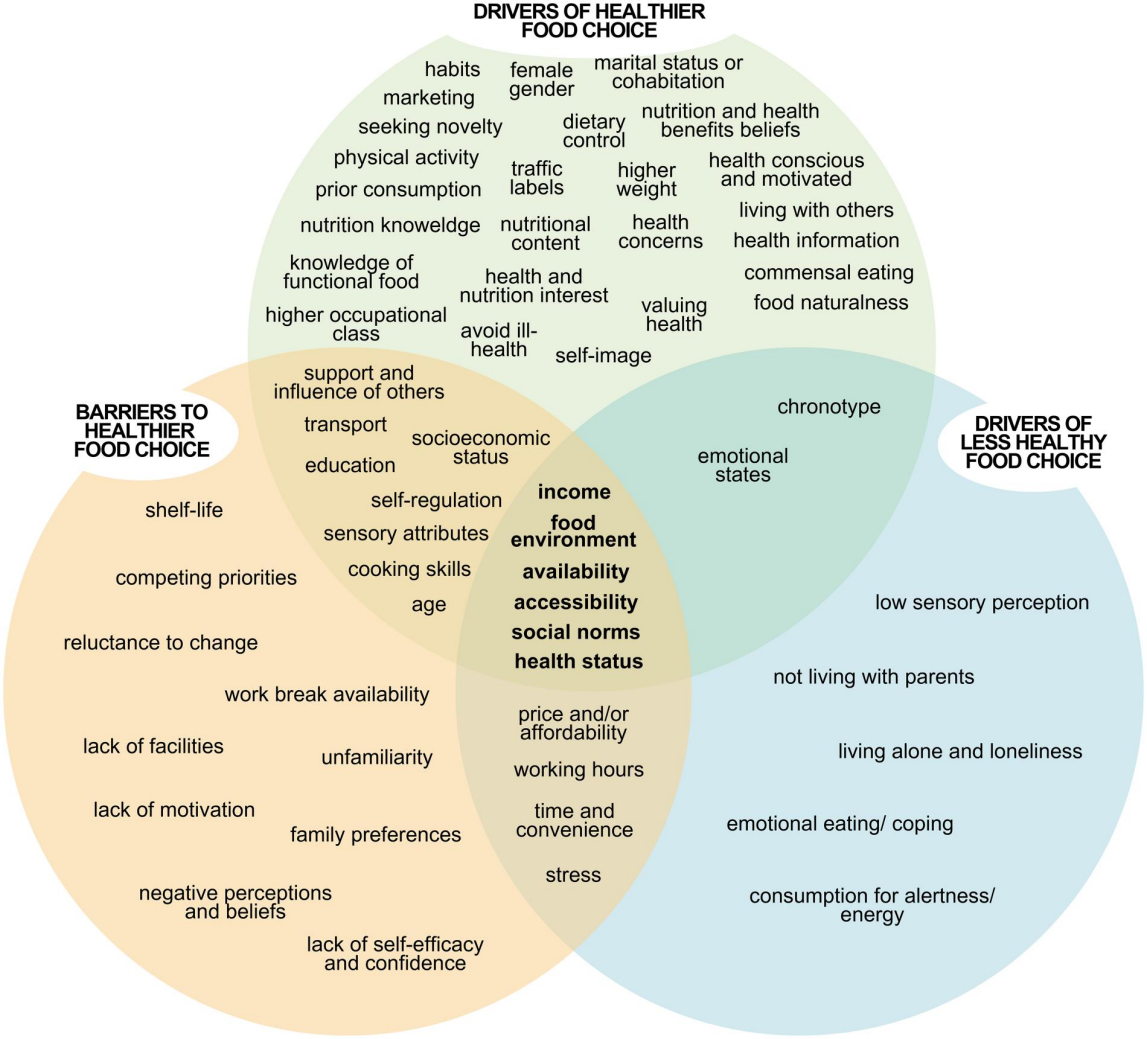
Similarities and Differences in Drivers of Food Choice

Figure 2 outlines the similarities and differences in the drivers of healthier and less healthy food choice, and barriers to healthier food.

##### Similarities across drivers

Several drivers were reported as both drivers and barriers to healthier eating, in addition to being drivers of less healthy eating. These include income, availability, accessibility, social norms, health status and the food environment. This suggests these have a multi-faceted influence on food choice. Within these, having higher income, availability, and accessibility of healthy food, supportive social norms, and a healthier food environment are drivers of healthier eating while the opposite are described as barriers to healthier eating and drivers of less healthy eating. Regarding health status, having both good and poor health are described as driving healthier eating whereas poor physical health, function or dental incapabilities (eg, difficulty chewing) encourage less healthy eating and create barriers to healthier eating. There are some instances of drivers being reported more frequently in one direction than another. For example, availability is reported as a barrier among 13 of the included reviews, while this is reported as a driver of healthier eating among just five.

Both chronotype and emotional states drive healthier and less healthy food choice in symmetrical, but opposite ways. For example, morning chronotype, or positive emotional states positively influence healthier eating, whereas evening chronotype or negative emotional states influence toward less healthy eating.

##### Differences in drivers

Some drivers were found to not overlap. For example, a lack of facilities, family preferences, lack of motivation and competing priorities are reported as barriers to healthier eating. However, these were not reported as drivers of healthier food choice (ie, the presence of facilities was not reported as a driver of healthier eating). Furthermore, there were other drivers which were reported uniquely in the context of healthier, or less healthy food choices. Having health concerns and having an interest in health and nutrition were reported as drivers of healthier food choice, while living alone and emotional eating were reported as drivers of less healthy food choice.

While the findings here are limited by the included literature and cannot be taken as a comprehensive list of all drivers of food choices, the findings suggest there may be some differences, as well as similarities in drivers for different food types.

### RQ2(1). Types of Food

Within this review of reviews, several reviews considered particular types of food which enabled a low-level granular exploration of the subgroup similarities and differences in drivers of food choice (**Table 5**). In terms of particular types of food, nine reviews focused on healthy food and dietary behavior^8^^,^^26^^,^^40^^,^^47^^,^^63^^,^^68^^,^^71^^,^^72^^,^^85^ one considered both healthy and unhealthy food outcomes,^28^ five reviews focused on functional, novel, or modified food,^33–36^^,^^41^ and two reviews focused on seafood.^48^^,^^53^ Overlapping drivers include higher education, income, older age, physical activity, nutrition knowledge, positive sensory attributes, and nutritional and health beliefs. Reviews focused on functional, novel, or modified food report prior consumption to drive consumption, while valuing health and healthy eating is reported for healthy food generally, and higher socioeconomic status for seafood. There are no overlapping barriers for these three food types. Barriers reported by included reviews focused on particular food types include unfamiliarity (functional, novel or modified food), working hours (healthy food generally), and family preferences (seafood).

**Table 5.** Sub-Analysis of Drivers by Type of Food

Category	Drivers of healthier food	Food type	Barriers to heathier food	Food type
Demographic Drivers	Female gender	FNM; H	Higher education	FNM; H
	Higher education	FNM; H; S	Lower income	
	Higher income	FNM; H; S	Lower SES	H
	Higher occupational class	H; S	Younger age	FNM; H
	Higher socioeconomic status	S		
	Older age	FNM; H; S		
Environmental Drivers	Accessibility		Accessibility	H
	Availability	H; S	Availability	H; S
	Healthier food environment		Food environment	
	Marketing	FNM; H	Price / affordability	H; S
			Lack of facilities	H
Household and Lifestyle	Habits	H; S	Family preferences	S
	Physical activity	FNM; H; S	Time and convenience	H
	Prior consumption	FNM	Transportation	
	Seeking novelty	FNM; S	Working hours	H
	Living with family or others		Work break availability	H
	Access to transport		Reluctance to change	
	Marital status or cohabitation			
Knowledge and Skills	Cooking skills	H; S	Lack of cooking skills	H; S
	Knowledge of functional food	FNM		
	Nutrition knowledge	FNM; H; S		
Physical Characteristics	Avoid ill-health	S	Poor health or physical function	S
	Benefits to self-image	H		
	Good Physical Health	H		
	Health problems	FNM		
	Higher weight or BMI	FNM; H		
	Morning chronotype			
Product Characteristics	Food naturalness	H	Shelf-life	H
	Health information	FNM; H	Undesirable sensory attributes	H; S
	Nutritional content	FNM; H		
	Positive sensory attributes	FNM; H; S		
	Traffic light labels	H		
Psychological Drivers	Dietary control	H	Competing priorities	H
	Health and nutrition interest	H	Lack of motivation	H
	Health concerns	FNM; H	Stress	H
	Health conscious and motivated	FNM; H	Unfamiliarity	FNM
	Nutritional and health benefit beliefs	FNM; H; S	Lack of self-control or regulation	H
	Positive emotional states	H; S	Lack of self-efficacy and confidence	H; S
	Self-regulation	H	Negative perceptions and beliefs	FNM; S
	Valuing health and healthy eating	H		
Social Drivers	Commensal eating		Negative influence of others	H
	Community, family and peer support	H	Social norms of acceptability	H
	Social norms	H; S		

Abbreviations: BMI, body mass index; H, Healthy Food (General); FNM, Functional, Novel or Modified Food; S, Seafood.

### RQ2(2). Financial Drivers

Eleven reviews noted that lower income, higher price of healthier food relative to less healthy food, and affordability were all barriers to healthier eating,^8^^,^^26^^,^^33^^,^^48^^,^^50^^,^^53^^,^^62^^,^^66^^,^^73^^,^^77^^,^^82^ with lower socioeconomic groups noted to face greater barriers in accessing healthier food.^8^^,^^85^ Four reviews noted that affordability, low prices of less healthy food, or high prices of healthier food, were drivers of less healthy choices.^28^^,^^52^^,^^75^^,^^80^ Two reviews noted that low income was a driver of less healthy eating.^28^^,^^76^ University and workplace environments were reported to present students and workers with higher costs of healthier compared with less healthy food options.^45^^,^^47^^,^^64^^,^^67^^,^^68^^,^^71^

Five reviews discussed an association between higher income or socioeconomic status and healthier food choices.^28^^,^^53^^,^^58^^,^^80^^,^^85^ Two reviews noted that low in-store prices or the perception of value for money can drive healthier food choices.^66^^,^^82^ Furthermore, one review noted that improvements in income can protect consumers against negative impacts of drivers of food choice.^72^ Finally, two reviews reported age to be associated with willingness to pay for functional food.^35^^,^^36^

### Drivers by Age Group

Within the included reviews, seven reviews explored determinants among students and/or young people^26^^,^^43^^,^^45^^,^^64^^,^^65^^,^^67^^,^^78^ and six reviews focused on older adults.^28^^,^^52^^,^^58^^,^^76^^,^^79^^,^^86^ It was not an explicit aim of this review to explore differences in drivers occurring by age group, however there was an opportunity to review this for the included literature. There are some overlapping drivers of healthier food choice for younger and older adults. These are being female, having habits related to healthy eating, living with family or others, being health conscious and motivated, positive emotional states and valuing health and healthy eating. There are also some drivers of food choice which were not overlapping among the included reviews such as the desire to avoid ill-health and having a health or nutrition interest (older adults), and benefits to self-image and social norms (younger adults and students). Overlapping drivers of less healthy food choice are: a food environment driving less healthy food choice, and price/ affordability. Accessibility is the only overlapping barrier to healthier food choice. These findings suggest some similarities (generalisable across age groups) and possible differences (unique to particular age groups) in drivers of healthier and less healthy food choices.

## DISCUSSION

### Summary of Findings

The most frequently reported drivers of less healthy food choice were price and/or affordability, stress, and the availability of less healthy food alongside the lack of availability of more healthy food. For healthier food choice, these were being health conscious and motivated, nutrition knowledge, community, family and peer support, female gender, higher education and older age. Frequently researched barriers to healthier food choice were price and/or affordability, availability, a lack of time and need for convenience, accessibility, and undesirable sensory attributes of healthier food. Few included reviews reported barriers to less healthy eating. However, Hill and colleagues^32^ noted that the stress-unhealthy eating relationship could be weakened with higher dietary restraint. Additionally, improvements in both income and education, healthy habits and cooking skills, can protect people against negative influences on food choice (eg, problems around availability or the transition to university).^67^^,^^72^ Thus, dietary restraint, income, education, healthy habits, and cooking skills may be considered as barriers to less healthy food consumption.

This review of reviews showed some mixed findings in relation to the food environment. Some reviews reported largely nonsignificant or inconsistent relationships between the food environment and food choices^38^^,^^44^ while others reported findings in general alignment with Sawyer and colleagues^10^ who determined that the food environment results in a trend toward increased availability and affordability of less healthy foods; seen in particular among reviews situated in work and university contexts.^45^^,^^47^^,^^64^^,^^71^ The findings of this review align with other research suggesting importance of time and convenience as a factor influencing people toward less healthy food choices. Wider literature has reported a shift toward convenience food consumption driven in part by the value placed on time by consumers, and that a perceived lack of time can be a barrier to healthier eating.^89–91^

The review found similarities and differences in drivers influencing food choice for healthier versus less healthy food, showing an asymmetry in the literature and a more granular understanding of food consumption (Figure 2). This may signal some differences in the drivers influencing healthier versus less healthy food and is supported by research^25^ which has cautioned against assuming that an absence of drivers of healthier eating are therefore factors which drive less healthy eating.

The analysis of drivers by particular food types among included literature suggests different drivers of food choice are relevant within different groups of food. This should be considered when tailoring interventions to support an increase in particular types of food eg, when planning an intervention to increase seafood intake, family preferences may be of particular concern. Furthermore, while it was not an aim of this review to examine drivers by age group, findings suggest there may also be differences in drivers for different age groups. For example, younger adults may benefit from interventions which support them alongside others in their social environments as social norms appeared to be a relevant factor influencing food choice for this group. Conversely, older adults may benefit from interventions which focus on the benefits of healthy eating to maintain good health and avoid ill-health. Further research examining this specifically may be of use.

With regards to financial drivers, a bi-directional impact of price, affordability, and income was reported among the reviews, whereby higher income was associated with healthier food choices while lower income, lower price of unhealthy, and higher prices of healthy food were all associated with less healthy food choices. Research has shown an asymmetry in consumer response to price changes, with a tendency toward loss aversion, and higher sensitivity to price increases (resulting in losses for the consumer) than price decreases (gains).^92^ This review of reviews aligns with this evidence as price/affordability was the most frequently reported barrier to healthier food choice. Higher prices of healthier food may result in losses, which have a greater influence on food choice as a barrier. The relatively higher frequency of reporting of this barrier may point to an inequality in the evidence base or may indicate that this is a significant barrier. Despite this finding, some research disputes whether price and food affordability are objective or perceived barriers^8^ and there is conflicting research as to whether healthier food is more expensive than less healthy food.^93^^,^^94^ A similar question is raised for other drivers, such as whether time, or access, are perceived or objective barriers to healthier food choice.^8^^,^^46^ Future research into this area may be fruitful to distinguish objective from perceived drivers influencing food choice and where interventions can overcome these.

The suggestion of overlapping and distinct drivers of food choice have been reported elsewhere.^24^^,^^25^ A key strength of this review of reviews is the implications of these findings and their importance to policy and intervention design. Interventions should be tailored to target the most relevant drivers of food choice in both directions. For instance, despite nutrition knowledge being an important driver of healthier food choice, there is evidence that interventions targeting this alone are not greatly effective in comparison to more intrusive interventions which target external determinants.^20^^,^^95^^,^^96^ It may be more effective to implement interventions which tackle causes of less healthy eating (eg, stress, price) in combination with improving nutritional knowledge to support dietary changes in both directions. Furthermore, this review of reviews highlights the complexity in drivers, which are diverse at the individual level (eg, chronotype), but also for different groups (eg, younger vs older), and for different food types (eg, functional food). The multiple layers of influence sit alongside intersectionality of individuals to suggest that a person-centred approach to supporting healthy eating is also required.

Some reviews have noted the interrelated nature of drivers,^28^^,^^41^^,^^76^ and the way that combinations of drivers may have shifting influences as they dynamically interact. For instance in absence of eating habits some factors, such as the negative influence of peers or the ease of convenience foods, may exhibit a greater influence on food choice.^67^ Similarly, the presence of less healthy foods may outweigh a person’s self-control.^8^ Other difficulties in determining the relative importance of drivers are that trade-offs between drivers are not captured eg, convenience vs quality.^86^ Future work may benefit from a focus on this interaction between drivers.

### Strengths and Limitations

The limitations emphasised among the reviews included the cross-sectional nature of evidence, the heterogeneous nature of methods and the lack of reliability or validation of some data collection measures. Concerns of reverse causality associated with cross-sectional research have been voiced.^83^ For example, rather than availability resulting in higher consumption of healthier food, individuals with healthier diets may choose to shop where there is high availability of these products. In line with this, Walker-Clarke and colleagues^86^ reported higher emotional eating among older adults with obesity, signalling the possibility of reverse-causality or bi-directionality of some drivers and the food consumption outcomes. This highlights the need for longitudinal research in this field to enable clarification about the direction of causality.

The CASP checklist for systematic reviews was chosen as included reviews were required to have core components of a systematic search. It is acknowledged, however, that this may have excluded relevant reviews which did not have these elements. This review of reviews sought to examine the evidence on drivers of food choice among adults in high income countries. Synthesised findings were not always segregated by sample age or country income, thus limiting the ability of the authors to do this. Nevertheless, review results relating to excluded populations or settings were ignored where specified.

Reviews differed in whether they looked at drivers of particular food types or broader dietary behaviors. Consuming less healthy foods can fit into a broader healthy diet (and vice versa) and it is unclear here if there are differences between drivers of healthier (less healthy) foods vs diets. Only 37% of included reviews provided definitions relating to food, limiting clarity about how different authors categorised foods as healthier or not. Finally, this review of reviews is limited by the scope of included research and caution should be taken with interpretation of the findings.

### Conclusions and Future Research

This review of reviews sought to examine the similarities and differences in drivers of healthier versus less healthy food choices. Overall, a lack of clarity throughout the literature in how healthier and less healthy foods and diets are described was evident which future research should seek to rectify. In total, 59 reviews were included, with findings suggesting differences as well as similarities in the drivers reported for healthier and less healthy food choices. This contributes to the literature through a more nuanced understanding of drivers and supports intervention approaches which target drivers increasing healthier and decreasing less healthy eating. Other areas for future research to explore include understanding the difference between perceived and objective drivers, gathering longitudinal research in this field, and investigating the relative importance of drivers while acknowledging the dynamic interplay between them.

## Supplementary Material

nuaf202_Supplementary_Data
