# Supplementary material for: Do the Drivers of Food Choice Differ Between Healthier and Less Healthy Foods? A Systematic Review of Reviews
Source: Nutr Rev. 2025 Nov 10;84(8):1673–95. doi: 10.1093/nutrit/nuaf202 (PMC13353532; doi:10.1093/nutrit/nuaf202)
Supplement: nuaf202_Supplementary_Data [file nuaf202_supplementary_data.zip › Supplementary Materials v4.docx]

# Supplementary Materials

## Additional File 1a – Search Strategy

**OVID (Embase, PsycINFO, MEDLINE)**

1 (Drive* or cause* or factor* or determinant* or influenc* or correlat* or underlie* or underlying or barrier* or facilitator* or motiv* or constraint* or choice* or behaviour* or behavior*).ti,ab

2 ((Food* or diet* or eat* or meal*) adj3 (health* or unhealth*)).ti,ab.

3 ((systematic or literature or umbrella) adj3 (review* or overview*).ti,ab.

1 AND 2 AND 3

**SCOPUS**

1 (Drive* or cause* or factor* or determinant* or influenc* or correlat* or underlie* or underlying or barrier* or facilitator* or motiv* or constraint* or choice* or behaviour* or behavior*) title, abstract

2 ((Food* or diet* or eat* or meal*) W/3 (health* or unhealth*)) title, abstract

3 ((systematic or literature or umbrella) W/3 (review* or overview*)) title, abstract

1 AND 2 AND 3

**Econlit**

AB (Drive* OR cause* OR factor* OR determinant* OR influenc* OR correlat* OR underlie* OR underlying OR barrier* OR facilitator* OR motiv* OR constraint* OR choice* OR behaviour* OR behavior*) AND AB ( (Food* OR diet* OR eat* OR meal*) N3 (health* OR unhealth*) ) AND AB ( (systematic OR literature OR umbrella) N3 (review* OR overview*) )

## Additional File 1b – Search Terms Relating to Drivers of Healthier and Less Healthy Food.

|  | Drive* / cause* / factor* / determinant* / influenc* / correlat* / underlie* / underlying / barrier* / facilitator* / motiv* / constraint* / choice* / behaviour* / behavior* |
| --- | --- |
| AND | Food* / diet* / eat* / meal* |
| NEAR | Health* / unhealth* |
| AND | Systematic / literature / umbrella review* / overview* |

## Additional File 1c – Table of Extracted Findings from Included Reviews

| **Author & Year of Publication** | **Driver of Healthier Eating** | **Driver of Less Healthy Eating** | **Barrier to Healthier Eating** |
| --- | --- | --- | --- |
| **Alsubhi et al. (2022)** | **WTP** for healthier food products, particularly low-fat products |  | Younger consumers |
|  | European consumers have higher **WTP** for healthy food products vs other countries |  | Higher levels of education and **income** |
|  | Older age (60+) |  |  |
|  | Female gender |  |  |
|  | Being overweight or obese |  |  |
|  | Having health concerns |  |  |
|  | Regular physical activity |  |  |
| **Baker et al. (2022)** | Socio-demographic characteristics (**willingness to pay premium when associated with improvements in household health**) |  | Psychological characteristics (perceived risks / unfamiliarity) |
|  | Product characteristics (effective branding, health information on labels, taste, acceptance of healthy carrier) |  |  |
|  | Psychological characteristics (health consciousness, knowledge of functional food, acceptance of health benefits, trust/ credibility in advertisement) |  |  |
|  | Physical characteristics (diet-related health problems, higher BMI, poor subjective health, health complaints) |  |  |
|  | Behavioural characteristics (healthy lifestyle / health-promoting behaviour, previous consumption of functional food, novelty products) |  |  |
| **Barlow et al. (2016)** | Interventions targeting reduced discount rates | Higher discount rates |  |
|  |  | Diets rich in sugary foods |  |
| **Bennett et al. (2022)** | Higher education |  |  |
|  | Higher occupation level |  |  |
| **Bernardo et al. (2017)** |  | Students who did not live with their parents had less healthy eating habits than those who did, after entered at university |  |
| **Bimbo et al. (2017)** | Female consumers | ‘Unnatural’ match between carrier and ingredient | ‘Unnatural’ match between carrier and ingredient |
|  | Willingness to use/ **purchase** increases with age |  |  |
|  | Diet-health and nutritional knowledge |  |  |
|  | Natural’ match between carrier and the bioactive ingredient |  |  |
| **Bivoltsis et al. (2023)** | (Some evidence) healthier community food environment |  |  |
| **Caruso et al. (2023)** |  | Convenience (time constraint) | Lack of nutritious options in the campus food environment |
|  |  | **Affordability** (budget constraint) | **Cost** of more nutritious food options relative to less nutritious options |
|  |  |  | Poor quality / taste of nutritious options |
|  |  |  | All-you-can-eat dining services |
| **Caso & Vecchio, (2022)** | Situational factors (healthy habits, live alone/ with others, use of natural ingredients, media/ advertisement, health self-monitoring, community support) | Situational factors (live alone/ with other, unhealthy food habits, food outlets in area, lack of food programme, loss of partner, fewer stocked outlets) |  |
|  | Psychological factors (health and nutrition interest, positive and motivated, low familiar products/ children memories) | Psychological factors (food fussiness, lower health and nutrition interest, low familiar products/ children memories) |  |
|  | Biological and physiological factors (good physical and function activity, female, old age) | Biological and physiological factors (physical and dental incapabilities, low appetite /forgetting to eat, health problems) |  |
|  | Sociocultural factors (diet knowledge/ cooking skills, occupation class, country/ area, high education, **high income**, land/ back garden) | Sociocultural factors (low diet knowledge/ cooking skills, country/ area, low education, **low income**, no personal transport) |  |
|  | Extrinsic product characteristics (certification/ health claim) | Extrinsic product characteristics (difficulty understanding claim, portion size/ **offerts, price**) |  |
|  | Intrinsic product characteristics (texture, little attention on sensory attributes) | Intrinsic product characteristics (texture, low sensory perception, low availability of fresh products) |  |
| **Caspi et al. (2012)** | Availability associated with healthy diet |  |  |
| **Cheong et al. (2022)** | (Personal) Family support motivates and increases confidence in healthy eating. |  | (Personal barriers) Not having the goal to eat healthily; Prioritisation of commitments above healthy eating; Lack of motivation; Lack of self-confidence; Lack of self-control; The environment not conducive to healthy eating. Nurses influenced by people who ate unhealthily. |
|  | (Interprofessional and patient-related) Healthy eating can become the norm. |  | (Interprofessional and patient-related barriers) Culture / dietary norm of consuming unhealthy food; Lack of breaks. |
|  |  |  | (Organisational barriers) Difficulty finding **affordable** healthy meals in hospitals; Inadequate storage facilities, fatigue, long and stressful shifts; Confectionaries eaten to stay alert. |
| **Christenson et al. (2017)** | Perceived health benefits |  | **Price** |
|  | Taste |  | Availability |
|  | Convenience |  | Concerns about quality |
|  | Change from meat |  | Lack of confidence |
|  | Habit |  | Preferences of family members |
| **De Cianni et al. (2022)** | Consumer attributes (knowledge of nutritional and medicinal value) |  | Food neophobia |
|  | Product attributes (flavour, taste) |  | **Price** |
|  | **Willingness to pay** when cost equal to alternatives |  |  |
|  | Health and sustainability information |  |  |
| **De Steur et al. (2015)** | Higher acceptance in US vs other countries |  | Lower **WTP premium** if student (younger, higher education), anti-GM information |
|  | **Willingnes to pay** 25-70% for GM products with higher vitamin levels |  | Lower preference for GM over regular foods if student (young, high education) |
|  | Higher **WTP premium** if non student (low education), regular consumption, knowledge of **regular price,** knowledge of GM technology, acceptance of GM technology, information about nutritional benefits and regional situation) |  |  |
|  | Preference for GM over regular foods if non student (older, low education), regular exercise |  |  |
| **Devonport et al. (2019)** |  | Stress |  |
|  |  | Emotional eating under stress |  |
| **Enriquez & Archila-Godinez, (2022)** | Education level | Emotions e.g., excited, stressed, hungry and being in an environment with smells of high-calorie foods |  |
|  | Higher cultural capital |  |  |
|  | Food packages from food banks |  |  |
|  | Nutritional information |  |  |
|  | Desire to improve and maintain health |  |  |
| **Eskandari et al. (2022)** |  | Charitable sources e.g., food banks | **Low income** |
|  |  |  | **Affordability of food** |
|  |  |  | Purchasing options |
|  |  |  | Accessibility |
|  |  |  | Non-perishable food items |
|  |  |  | Belief to be less nutritious |
|  |  |  | Transportation |
|  |  |  | Abundance of unhealthy food |
|  |  |  | Community programmes |
|  |  |  | Perceived unhealthy |
|  |  |  | Poor quality of food of food banks |
|  |  |  | Accessibility and **affordability** |
|  |  |  | Children’s preferences |
| **Fuente Gonzalez et al. (2022)** |  | Emotional eating |  |
|  |  | Stress, boredom and depression |  |
| **Govindaraju et al. (2022)** | Health status | Nutrition messaging (misleading) | Childhood experiences e.g., lower FV consumption and lower economic status |
|  | Avoid disability | **Cost of healthy food** | Physical functioning |
|  | Health maintenance | Food claims and communication of diet restrictions from professionals | Physical ability to cook |
|  | Maintain independence |  |  |
|  | Cooking skills |  |  |
|  | Knowledge |  |  |
|  | Social networks and connectedness |  |  |
|  | Past habits |  |  |
| **Govzman et al. (2021)** | Older age |  | **Price** (particularly for those on a lower income) |
|  | **Higher income** |  | Sensory or physical characteristics of seafood |
|  | Higher education |  | Household preferences |
|  | Employment level |  | Perceived risks |
|  | **Socioeconomic status** |  | Environmental barriers |
|  | Better knowledge of recommendations for fish |  | Lack of cooking skills |
|  | Nutritional knowledge |  | Negative psychological states/ personality traits |
|  | Physically active |  | Dislike of taste/ smell |
|  | Less likely to smoke |  | Dislike of texture |
|  | Personal preference |  | Physical impairments |
|  | Availability of fresh seafood |  | Lack of availability of fresh or good quality seafood |
|  | Cooking skills and knowledge |  |  |
|  | Health and nutritional beliefs |  |  |
|  | Positive psychological states or personality traits |  |  |
|  | Perceived health benefits |  |  |
|  | Belief that fish is healthy and contains important nutrients |  |  |
|  | Environmental influences (family, friends, social norms) |  |  |
|  | Increased risk of health issues |  |  |
|  | **Cost benefit** change with age |  |  |
|  | Good availability of fresh or good quality seafood |  |  |
| **Gupta et al. (2018)** | Dissemination of information | Liking of sugary food |  |
|  |  | Attitudes of peers |  |
| **Gupta et al. (2019)** | Health motivation | Night shift workers report more snacking | Difficulty bringing food from home |
|  | Avoid gastric upset | Vending machines | Social barriers |
|  |  | Purchasing take-aways | Long working hours |
|  |  | Unhealthy food in break areas | Break availability |
|  |  | Staying alert | Eating with colleagues |
|  |  | Stress |  |
| **Hanna et al. (2015)** |  | Living alone, particularly for lower FV and fish | Persons living alone were less likely to follow healthy diets |
| **Hanna et al. (2023)** |  | Loneliness or social isolation associated with lower FV, higher energy-dense nutrient-poor foods and lower overall diet quality |  |
| **Hill et al. (2022)** |  | Stress |  |
| **Host et al. (2016)** | Positive view of own health | Compensation for flavour loss | Poor dentition, dentures and difficulty with chewing |
|  | Interest in health and/or nutrition | Living alone | Will to remain independent and reluctance to accept help from others |
|  | Valuing good health | Psychosocial aspects | Male gender |
|  | Higher levels of physical activity | Physiological changes associated with aging | Limited access to food |
|  | Personal transport |  | Personal resources |
|  | **Higher disposable income** |  |  |
|  | Support from family, friends, government and community services |  |  |
|  | Knowledge and skills related to nutrition |  |  |
|  | Selection of appropriate foods |  |  |
|  | Adequate cooking techniques |  |  |
|  | Female gender |  |  |
|  | Personal resources |  |  |
|  | Psychosocial aspects |  |  |
| **Kazmierski et al. (2021)** | Stress (African Americans) | Stress (African Americans and Hispanic/Latinx) |  |
|  | Stress (Hispanic/Latinx) | Stressors (Non-Hispanic Whites) |  |
|  |  | Global perceptions of stress (African Americans) |  |
|  |  | Discrimination-related stressors |  |
|  |  | Uncontrollable stressors |  |
|  |  | Overall perceptions of life stressfulness, emotional difficulty of life and difficulty coping (Hispanic/Latinx) |  |
| **Khan & Pandey, (2023)** | Positive health motivations and goals | Negative emotions |  |
|  | Customisation of meals at food retailers |  |  |
|  | Traffic light calorie labels |  |  |
| **Khoshghadam & Rajabi, (2024)** |  | Food as a coping mechanism: need to manage negative emotional states. |  |
|  |  | Food as reward: Gratifying nature of unhealthy food and rationalising unhealthy food consumption with indulgence. |  |
| **Kouritzin et al. (2023)** |  | Physical relocation to an urban neighbourhood | Lack of time |
|  |  |  | Competing priorities |
|  |  |  | Lack of accessible transportation |
|  |  |  | Lack of grocery stores within waking distance |
|  |  |  | Cost |
|  |  |  | Not adjusting habits to favour a healthy diet |
| **Li et al. (2019)** | Theory of Planned Behaviour variables |  |  |
|  | Intention the most proximal determinant of healthy dietary behaviours |  |  |
| **Li et al. (2022)** |  | The university food environment | Close availability of junk foods and **costly** FV options |
|  |  | The university food environment has low availability and **higher cost** of healthy food vs unhealthy options |  |
| **Lyzwinski et al. (2018)** |  | Stress | Stress |
| **Madlala et al. (2023)** | Access to supermarkets, large grocery stores and farmers markets | Greater access to fast food restaurants | **Cost** |
|  | Lower in-store food prices |  | Transportation |
|  | Food assistance programmes |  | Limited access |
|  | Access to transportation |  | Presence of unhealthy food stores |
|  | In-store availability |  | Convenience and corner stores |
|  | Quality of food |  |  |
|  | Marketing of healthy food |  |  |
| **Maillet & Grouzet, (2021)** | Social influence and social support (support from peers and parents) | Peers and parents | Transition to university |
|  | Social influence and social support (peers and parents who eat healthy and provide advice) | Changes in diet and eating habits (moving into residence or off-campus housing) | Lack of healthy options, presence of unhealthy food, convenience foods and absence of adequate cooking facilities |
|  | Social influence and social support (support from others fosters autonomous motivation) | Changes in diet and eating habits (losing regular eating patterns and routines) | University food environment not supporting psychological needs |
|  |  |  | Difficulty accessing **affordable healthy food** and avoiding salient and unhealthy foods |
|  |  |  | Ease of convenience foods, negative influence of peers, and university lifestyle |
|  |  |  | Social influence and social support (peers) |
|  |  |  | Little support for needs for autonomy, competence and interpersonal relatedness |
| **Marko et al. (2023)** | Considered roster planning |  | Perceived expectation of the role resulting in not taking breaks |
|  | Departmental role modelling |  | High availability and accessibility of unhealthy foods |
|  | Managers promoting healthy eating |  | **High cost** of healthy food |
|  | Supportive peer culture |  | Limited variety of healthy food |
|  | Allocating regular breaks |  | High reliance on vending machines |
|  |  |  | Eating on the go |
|  |  |  | Irregular working hours |
|  |  |  | Stress |
|  |  |  | Exhausting workload |
| **Mazri et al. (2020)** | Morning type | Evening chronotype |  |
| **McDermott et al. (2015)** | Perceived behavioural control – behaviour association |  |  |
| **Munt et al. (2017)** | Female gender |  | Male gender |
|  | Healthy diet of friends and family |  | Unhealthy diet of friends and family |
|  | Support/ encouragement of friends and family |  | Expected consumption of unhealthy foods in certain situations |
|  | Desire for improved health |  | Relative **cheapness of unhealthy foods** |
|  | Desire for weight management |  | Lack of time to plan, shop, prepare and cook healthy foods |
|  | Desire for improved self-esteem |  | Lack of facilities to prepare, cook and store healthy foods |
|  | Desire for attractiveness to potential partners and others |  | Widespread presence of unhealthy foods |
|  | Possessing autonomous motivation to eat healthy |  | Lack of knowledge and skills |
|  | Existence and use of self-regulatory skills |  | Lack of motivation |
|  | Increased planning, automaticity and habit of healthy eating |  | Lack of self-regulation behaviours |
|  | Social norms, foster inclusion |  | Preferred taste for unhealthy food |
|  | Having an incentive |  | Baseline hunger and lack of satiation |
|  |  |  | Emotional responses (e.g. stress) |
|  |  |  | Ease of access and availability of unhealthy diets |
|  |  |  | Convenience of unhealthy food |
| **Needham et al. (2019)** |  |  |  |
| **Nicholls et al. (2017)** | Being knowledgeable and motivated | Organisational factors (unfavourable work schedules, snacking on junk food due to perceived energising effects or emotional coping strategy) | Organisational factors (unfavourable work schedules, long working hours and shift work) |
|  | Features of the social and physical environment | Physical workplace environment (junk food is accessible and **cheaper than healthy alternatives**) | Individual factors (poor motivation and moderate self-efficacy related to healthy eating, inadequate nutrition knowledge, failure for some to recognise own weight status) |
|  |  | Social factors (unhealthy food from patients usually available) |  |
| **Ogundijo et al. (2022)** | Use of traffic light and warning labels on food | Unavailability of food products in nearby stores |  |
|  | Use of health and nutrition promotional messages |  |  |
|  | Healthiness of foods and consciousness of consumers |  |  |
| **Ojo et al. (2023)** | Community and religious groups | Accessibility of junk food in neighbourhoods | Time constraints |
|  | Nutritional knowledge | Dietary acculturation to host countries | **Costs** of healthy food |
|  | Desire for positive self-image | Accommodation of children’s food habits | Poor availability of healthy foods |
|  | Family and community support | Cultural and religious festivals | Abundance of unhealthy food |
|  | Growing old |  | Familiarity |
|  | Ill-health |  | Food preferences |
|  |  |  | Lack of cooking skills including healthy cooking skills |
| **Pinto et al. (2021)** | Sustainability, purity and naturalness |  | Satiety, liking, the need for taste and indulgence |
| **Pitt et al. (2017)** |  | Availability, accessibility and **affordability** |  |
| **Poggiogalle et al. (2021)** | Family structure and living situation (marital status, living with others, presence of neighbours) | **Lower income** |  |
|  | Higher education level |  |  |
| **Ravikumar et al. (2022)** | Cooking interventions | Food environment high in convenience stores and fast food outlets | Lack of time |
|  | Desire to provide family with healthy food | Busy schedule | **Financial constraints and cost** of food |
|  |  |  | Limited availability of fresh food |
| **Rodrigues et al. (2019)** | Being female |  |  |
|  | Normal weight |  |  |
|  | Living in the family home |  |  |
|  | Greater perception of happiness and less pressure and stress |  |  |
|  | Importance given for healthy eating |  |  |
| **Shahrin et al. (2019)** | Health importance |  |  |
| **Sobhani & Babashahi, (2020)** | Demographic factors (female gender) | Demographic factors (larger households, households with younger age composition) |  |
|  | Socioeconomic factors (**higher income,** higher occupational social class, higher education, higher socioeconomic status) | Socioeconomic factors (**price of healthy food** leads to unhealthy choices for lower-income families, lower socioeconomic status) |  |
|  | Environmental factors (urban area, more frequent trips and fewer small trips) |  |  |
| **Teixeira et al. (2022)** | Morning type | Late chronotype associated with low adherence to healthy diets |  |
|  |  | Eveningness |  |
|  |  | Late types present higher consumption of processed and ultra processed foods, sweets, food with added sugar, fast food and chocolate |  |
| **Tsofliou et al. (2022)** | Cognitive: Improved diet quality; Physical benefits; Appearance/ body weight benefits; Psychological benefits. |  | Availability / accessibility: Difficulty purchasing food items; Seasonal availability of foods; Limited access, and choices. |
|  | Demographic: Higher education; Female gender. |  | Cognitive: Absence of nutrition education; Lack of knowledge of how to incorporate MedDiet foods; Complex dietary info; Low health appeal and perceived healthiness;  Negative perceived outcomes; Nutritional attitudes and beliefs. |
|  | Financial: Good **value for money.** |  | Demographic: Lower education; Younger age. |
|  | Motivational: High self-efficacy/ self-determination; Good self-regulation; Higher stage of dietary change. |  | Financial: Increased **food costs;**  Wasteful/ foods easily spoil; **Lower income.** |
|  | Sensory and hedonic: Enjoyable and pleasurable eating experience; High taste/ sensory appeal; Varied diet; Familiarity with foods. |  | Lifestyle: Habit of smoking; Medical concerns / poor health. |
|  | Socio-cultural: Being married or cohabitating; Family/ friend support; eating together. |  | Motivational: Lack of willpower / motivation to cook healthy foods; Resistance to dietary change. |
|  |  |  | Sensory and hedonic: Low sensory appeal; Not liking the taste/ smell;  Components of the diet were unappealing. |
|  |  |  | Socio-cultural: Negative influence of family and others; Usual habits; Acceptability of MedDiet – difficulty adapting to new eating pattern;  Stress, stressors, work and time pressures; Impractical; Cultural differences; Colder climate; Not convenient; Lack of cooking skills / equipment; Time to plan, purchase and prepare foods. |
| **Turner et al. (2021)** | Fruit and vegetable access in the retail food environment |  |  |
|  | Fruit and vegetable availability in the retail food environment |  |  |
| **van der Merwe et al. (2022)** | Morning type | Evening type |  |
|  |  | Late chronotype |  |
| **Vilar-Compte et al. (2021)** | **Higher socioeconomic status** associated with better access to healthy food in urban areas | **Food insecurity** | **Lower socioeconomic status** associated with poorer access to healthy food in urban areas |
|  |  |  | **Price of healthy food** higher in poor neighbourhoods with fewer food retail stores |
|  |  |  | **Urban poverty** |
| **Walker-Clarke et al. (2022)** | Perceived dietary control (internal locus of control and high sense of self-efficacy) | Mental health and mood (poorer mood and mental health) | Food decision making (control over food choice, resistantance to change) |
|  | Mental health and mood (better mood and mental health) |  | Eating arrangements (eating alone) |
|  | Eating arrangements (commensal eating increased dietary diversity) |  |  |
| **Yamaguchi et al. (2022)** | Healthy perceived food environment |  |  |
| **Zanchini et al. (2022)** | Lifestyle factors (physical activity correlated with interest and **willingness to pay**) |  |  |
|  | Lifestyle factors (higher BMI linked to interest in health information and **willingness to pay**) |  |  |
|  | Lifestyle factors (clinical family history concerns) |  |  |
|  | Psychological factors (knowledge) |  |  |
|  | Psychological factors (beliefs about nutritional quality, content and benefits of functional foods associated with **willingness to pay**) |  |  |
|  | Socio-demographic factors (women) |  |  |
|  | Socio-demographic factors (educated people were more **willing to pay**) |  |  |
|  | Socio-demographic factors (middle-aged and older consumers had higher interest and **willingness to pay**) |  |  |
|  | Socio-demographic factors (**high income** slightly more related to **willingness to pay** or **intention to buy** functional foods, but **low incomes** have also been related to this) |  |  |
|  | Socio-demographic factors (families with children) |  |  |
| **Zorbas et al. (2018)** | Individual level factors (nutrition knowledge and skills, beliefs and attitudes, psychology, and habits) |  | Environmental layer of influence (**food price and affordability,** food availability, food characteristics, time, and convenience) |
|  | Social level factors (social networks (family, friends, peers, and coworkers) |  | Individual level factors (physiological preferences) |
|  |  |  | Social level factors (marketing of unhealthy foods, sociocultural acceptability of healthy diet) |
|  |  |  | Supermarket availability and access to healthy foods are greater barriers for **lower socioeconomic groups** than the general population |
|  |  |  | Transportation and geographical challenges in areas of **lower socioeconomic position** |

## Additional File 1d – PRISMA checklist

| **Section and Topic** | **Item #** | **Checklist item** | **Location where item is reported** |
| --- | --- | --- | --- |
| **TITLE** | | |  |
| Title | 1 | Identify the report as a systematic review. | Title |
| **ABSTRACT** | | |  |
| Abstract | 2 | See the PRISMA 2020 for Abstracts checklist. | NA |
| **INTRODUCTION** | | |  |
| Rationale | 3 | Describe the rationale for the review in the context of existing knowledge. | Introduction |
| Objectives | 4 | Provide an explicit statement of the objective(s) or question(s) the review addresses. | Introduction |
| **METHODS** | | |  |
| Eligibility criteria | 5 | Specify the inclusion and exclusion criteria for the review and how studies were grouped for the syntheses. | Methods |
| Information sources | 6 | Specify all databases, registers, websites, organisations, reference lists and other sources searched or consulted to identify studies. Specify the date when each source was last searched or consulted. | Methods |
| Search strategy | 7 | Present the full search strategies for all databases, registers and websites, including any filters and limits used. | Methods and Additional Files |
| Selection process | 8 | Specify the methods used to decide whether a study met the inclusion criteria of the review, including how many reviewers screened each record and each report retrieved, whether they worked independently, and if applicable, details of automation tools used in the process. | Methods |
| Data collection process | 9 | Specify the methods used to collect data from reports, including how many reviewers collected data from each report, whether they worked independently, any processes for obtaining or confirming data from study investigators, and if applicable, details of automation tools used in the process. | Methods |
| Data items | 10a | List and define all outcomes for which data were sought. Specify whether all results that were compatible with each outcome domain in each study were sought (e.g. for all measures, time points, analyses), and if not, the methods used to decide which results to collect. | Methods |
|  | 10b | List and define all other variables for which data were sought (e.g. participant and intervention characteristics, funding sources). Describe any assumptions made about any missing or unclear information. | Methods |
| Study risk of bias assessment | 11 | Specify the methods used to assess risk of bias in the included studies, including details of the tool(s) used, how many reviewers assessed each study and whether they worked independently, and if applicable, details of automation tools used in the process. | Methods |
| Effect measures | 12 | Specify for each outcome the effect measure(s) (e.g. risk ratio, mean difference) used in the synthesis or presentation of results. | N/A |
| Synthesis methods | 13a | Describe the processes used to decide which studies were eligible for each synthesis (e.g. tabulating the study intervention characteristics and comparing against the planned groups for each synthesis (item #5)). | N/A |
|  | 13b | Describe any methods required to prepare the data for presentation or synthesis, such as handling of missing summary statistics, or data conversions. | N/A |
|  | 13c | Describe any methods used to tabulate or visually display results of individual studies and syntheses. | Methods |
|  | 13d | Describe any methods used to synthesize results and provide a rationale for the choice(s). If meta-analysis was performed, describe the model(s), method(s) to identify the presence and extent of statistical heterogeneity, and software package(s) used. | Methods |
|  | 13e | Describe any methods used to explore possible causes of heterogeneity among study results (e.g. subgroup analysis, meta-regression). | N/A |
|  | 13f | Describe any sensitivity analyses conducted to assess robustness of the synthesized results. | N/A |
| Reporting bias assessment | 14 | Describe any methods used to assess risk of bias due to missing results in a synthesis (arising from reporting biases). | N/A |
| Certainty assessment | 15 | Describe any methods used to assess certainty (or confidence) in the body of evidence for an outcome. | N/A |
| **RESULTS** | | |  |
| Study selection | 16a | Describe the results of the search and selection process, from the number of records identified in the search to the number of studies included in the review, ideally using a flow diagram. | Results |
|  | 16b | Cite studies that might appear to meet the inclusion criteria, but which were excluded, and explain why they were excluded. | NA |
| Study characteristics | 17 | Cite each included study and present its characteristics. | Table 2 |
| Risk of bias in studies | 18 | Present assessments of risk of bias for each included study. | Results, Quality Appraisal |
| Results of individual studies | 19 | For all outcomes, present, for each study: (a) summary statistics for each group (where appropriate) and (b) an effect estimate and its precision (e.g. confidence/credible interval), ideally using structured tables or plots. | N/A |
| Results of syntheses | 20a | For each synthesis, briefly summarise the characteristics and risk of bias among contributing studies. | N/A |
|  | 20b | Present results of all statistical syntheses conducted. If meta-analysis was done, present for each the summary estimate and its precision (e.g. confidence/credible interval) and measures of statistical heterogeneity. If comparing groups, describe the direction of the effect. | N/A |
|  | 20c | Present results of all investigations of possible causes of heterogeneity among study results. | N/A |
|  | 20d | Present results of all sensitivity analyses conducted to assess the robustness of the synthesized results. | N/A |
| Reporting biases | 21 | Present assessments of risk of bias due to missing results (arising from reporting biases) for each synthesis assessed. | N/A |
| Certainty of evidence | 22 | Present assessments of certainty (or confidence) in the body of evidence for each outcome assessed. | N/A |
| **DISCUSSION** | | |  |
| Discussion | 23a | Provide a general interpretation of the results in the context of other evidence. | Results |
|  | 23b | Discuss any limitations of the evidence included in the review. | Strengths and Limitations |
|  | 23c | Discuss any limitations of the review processes used. | Strengths and Limitations |
|  | 23d | Discuss implications of the results for practice, policy, and future research. | Discussion |
| **OTHER INFORMATION** | | |  |
| Registration and protocol | 24a | Provide registration information for the review, including register name and registration number, or state that the review was not registered. | Methods |
|  | 24b | Indicate where the review protocol can be accessed, or state that a protocol was not prepared. | Methods |
|  | 24c | Describe and explain any amendments to information provided at registration or in the protocol. | N/A |
| Support | 25 | Describe sources of financial or non-financial support for the review, and the role of the funders or sponsors in the review. | Disclosure Statements |
| Competing interests | 26 | Declare any competing interests of review authors. | Disclosure Statements |
| Availability of data, code and other materials | 27 | Report which of the following are publicly available and where they can be found: template data collection forms; data extracted from included studies; data used for all analyses; analytic code; any other materials used in the review. | N/A |

*From:*  Page MJ, McKenzie JE, Bossuyt PM, Boutron I, Hoffmann TC, Mulrow CD, et al. The PRISMA 2020 statement: an updated guideline for reporting systematic reviews. BMJ 2021;372:n71. doi: 10.1136/bmj.n71

For more information, visit: <http://www.prisma-statement.org/>

## Additional File 1e – PRISMA checklist for Abstracts

| **Section and Topic** | **Item #** | **Checklist item** | **Reported (Yes/No)** |
| --- | --- | --- | --- |
| **TITLE** | | |  |
| Title | 1 | Identify the report as a systematic review. | Yes |
| **BACKGROUND** | | |  |
| Objectives | 2 | Provide an explicit statement of the main objective(s) or question(s) the review addresses. | Yes |
| **METHODS** | | |  |
| Eligibility criteria | 3 | Specify the inclusion and exclusion criteria for the review. | No |
| Information sources | 4 | Specify the information sources (e.g. databases, registers) used to identify studies and the date when each was last searched. | Yes |
| Risk of bias | 5 | Specify the methods used to assess risk of bias in the included studies. | Yes |
| Synthesis of results | 6 | Specify the methods used to present and synthesise results. | Yes |
| **RESULTS** | | |  |
| Included studies | 7 | Give the total number of included studies and participants and summarise relevant characteristics of studies. | No |
| Synthesis of results | 8 | Present results for main outcomes, preferably indicating the number of included studies and participants for each. If meta-analysis was done, report the summary estimate and confidence/credible interval. If comparing groups, indicate the direction of the effect (i.e. which group is favoured). | Yes |
| **DISCUSSION** | | |  |
| Limitations of evidence | 9 | Provide a brief summary of the limitations of the evidence included in the review (e.g. study risk of bias, inconsistency and imprecision). | No |
| Interpretation | 10 | Provide a general interpretation of the results and important implications. | Yes |
| **OTHER** | | |  |
| Funding | 11 | Specify the primary source of funding for the review. | No |
| Registration | 12 | Provide the register name and registration number. | No |

*From:*  Page MJ, McKenzie JE, Bossuyt PM, Boutron I, Hoffmann TC, Mulrow CD, et al. The PRISMA 2020 statement: an updated guideline for reporting systematic reviews. BMJ 2021;372:n71. doi: 10.1136/bmj.n71

For more information, visit: <http://www.prisma-statement.org/>
